# Concerted Electron-Ion Transport by Polyacrylonitrile Elucidated with Reactive Deep Learning Potentials

**DOI:** 10.1021/jacs.6c05078

**Published:** 2026-06-02

**Authors:** Rajni Chahal-Crockett, Michael D. Toomey, Logan T. Kearney, Yawei Gao, Joshua T. Damron, Amit K. Naskar, Santanu Roy

**Affiliations:** Chemical Science Division, 6146 Oak Ridge National Laboratory, Oak Ridge, Tennessee 37830, United States

## Abstract

Charge transport in polymers, such as polyacrylonitrile (PAN), is crucial for electronics and energy storage. For instance, PAN can transport cations e.g., Li^+^, by facilitating dynamic cation-nitrile coordination in batteries. However, little is known regarding the underlying role of complex reactive polymer configurations. Herein, we develop a deep-learning potential, trained on *ab initio* energies and forces of nonequilibrium reactive PAN configurations, to unravel the kinetics of PAN cyclization initiated by a nucleophile (OH^–^ dissociated from LiOH) attacking the terminal nitrile carbon. We find, based on the reaction free-energetics, rates, and charge analysis, that the nucleophile attack producing the first ring is the rate-limiting step, which subsequently triggers Li^+^-coupled electron transfer along the PAN backbone, causing ∼10^4^ times faster sequential ring-formation of the remaining nitriles. PAN’s extended configurations, where dipolar and H-bonding interactions are minimal, enable such rapid kinetics. By validating our computational findings with IR and NMR experiments, we establish a pathway for designing reactive polymers with enhanced charge transport for energy applications.

## Graphical abstract

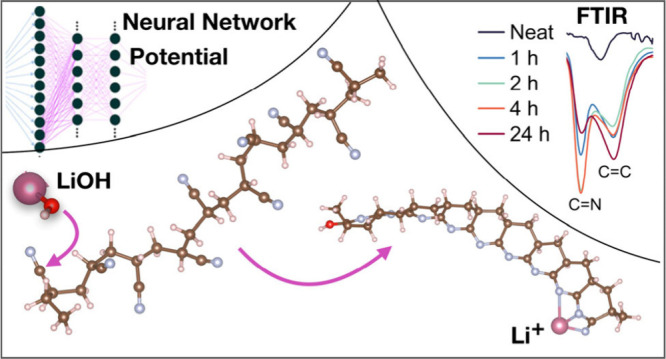

Charge transport, a fundamental event in condensed-phase systems, can be triggered via local molecular interactions,^1–3^ medium reorganization,^4–7^ and impurities or defects.^8–12^ Among many materials or molecules that exhibit charge transport events, polymers are particularly critical due to their broad range of applications, from organic electronics to energy storage. For example, redox-active polymers such as polyacetylene and polypyrrole are used to transport electrons through battery electrodes,^13–16^ while frequently used ion-conducting polymer electrolytes in batteries are poly­(ethylene oxide) and polyacrylonitrile (PAN).^17–23^ However, a major bottleneck for efficient charge transport by such polymers may arise from their complex diverse configurations. Several studies^24–26^ showed that more ordered and extended chain conformations can lead to faster electron transport due to enhanced electronic coupling between repeating units. Other studies^27–31^ indicate that amorphous, flexible polymer matrix can enhance ion diffusion. Nevertheless, the mechanism by which tunable polymer configurations can enable energy-efficient coupled electron–ion transport, critical for reaction-driven energy-storage systems,^32–35^ remains poorly understood.

Recently, the remarkable success^36–45^ in integrating deep-learning methods, electronic structure theory, and molecular dynamics (MD) simulations demonstrates the feasibility of accessing reactive chemistry, while exploring the large configurational space of a polymer. For instance, energies and forces obtained from density functional theory (DFT)-based *ab initio* MD of oligomers and their self-assembly can be leveraged to train a neural-network interatomic potential (NNIP).^46^ Subsequently, NNIP-based MD—referred to as NNMD—when combined with enhanced sampling techniques, such as umbrella sampling or metadynamics,^47–50^ provides potentially a powerful approach for investigating a wide range of reactive events, from local catalytic effects to cascades of bond-forming reactions in polymers. Herein, we demonstrate that an NNIP, trained on the DFT data from nonequilibrium reactive configurations enhanced-sampled along cyclization reaction paths of PAN, enables NNMD to examine the process of PAN-mediated electron-coupled ion transport.

PAN, as aforementioned, is a promising component of battery electrolytes with superior mechanical properties and electrochemical stability.^51–53,23,54^ It can leverage its nitrile (−C≡N) groups as coordination sites for cations, e.g., Li^+^ ions, guiding their transport within a battery through the polymer matrix. Herein, *both* computationally and experimentally, we showcase that PAN can be cyclized by an OH^–^ nucleophile attack at room temperature, which otherwise requires 200–300 °C.^55–58^ Such a cyclization process enables electron-coupled Li^+^ transport. As depicted in Figure 1, OH^–^ attacks C of a terminal −C≡N group, converting it to a −C=N^(−)^ (excess electron-localization at N) group, which again, as a nucleophile, attacks C of the adjacent −C≡N group, causing sequential cyclization up to the last unit and formation of a ladderlike configuration. Interestingly, Li^+^ moves along the cyclization steps, coordinating with the −C=N^(−)^ group, i.e., follows the propagation of the electron along the PAN backbone. By exploring reaction free-energy landscapes and employing transition state theory (TST), we find that such electron-coupled Li^+^ transfer can be effectively instantaneous in extended PAN configurations, provided the initial nucleophilic attack has already initialized. Our findings were corroborated through time-dependent spectroscopic measurements of the PAN-LiOH system dissolved in polar solvents.

**Figure 1:**
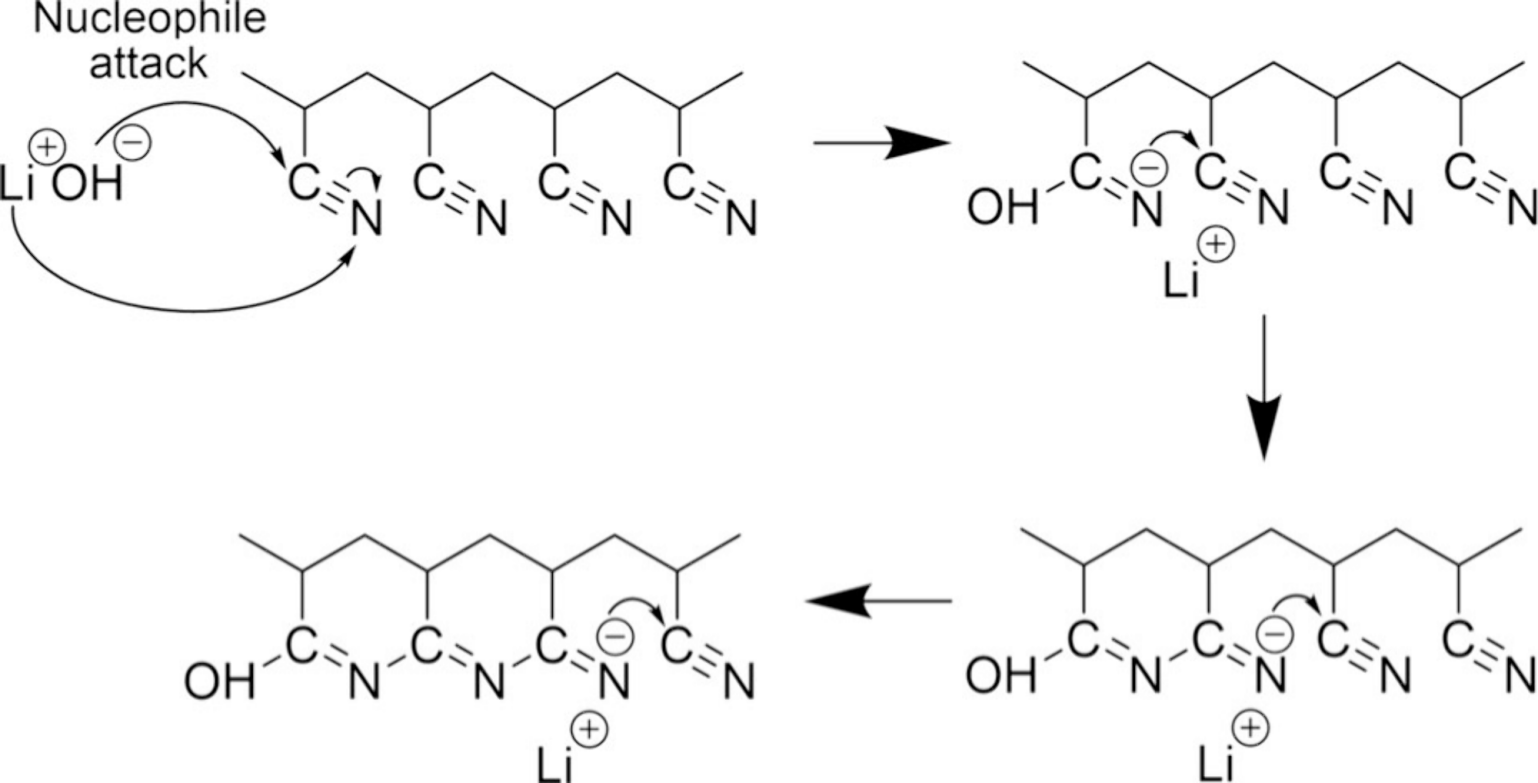
Mechanism of cyclization in a 4-unit PAN chain, initiated by the OH^–^ nucleophile. As the cyclization progresses, Li^+^ follows the electron localized on N, which again attacks the next −C≡N to continue the cyclization process.

Toward achieving quantitative understanding of the reaction free-energies and kinetics and the underlying configurational effects, we explore 8 generations (Gens 0–7) of NNIPs. Gen 0, developed in our earlier study,^46^ is suitable for simulating nonreactive PAN chains and bulk systems. As detailed in Supporting Information (SI), Gen 1 evolves to Gen 7, considering different reactions and configurational effects (Figures S1 and S2). The main consideration is to account for energies and forces from the AIMD simulations (detailed in SI) of the PAN configurations generated in the umbrella sampling of two reaction coordinates; the distance between the hydroxyl oxygen and C of the terminal −C≡N group (r_OC_) and the distance between N of a −C=N^(−)^ group and C of the adjacent −C≡N group (r_CN_) (Figure 1). The inclusion of other critical effects such as chain–chain dipolar and H-bonding interactions (considering uncyclized and partially cyclized chains) in dimeric and bulk configurations in the training ultimately leads to a robust, accurate, and stable NNIP, Gen 7 (Figures S3–S11), which is used for all final computations of reaction free-energies.

Figure 2 depicts the free-energy profiles of the cyclization steps for a 4-mer and a 10-mer chains in the gas-phase environment. We choose these oligomers to mimic local structures of an extended large polymer, avoiding astronomically high configurational barriers that may arise from strong −C≡N dipole–dipole interactions and −C≡N···HC– hydrogen bonding in folded, contracted, or coiled bulky structures (Figure S14) typically expected in the gas-phase environment. The free-energy profiles in Figure 2 predominantly reveal the barriers for a combined effect of bond rotation, moderate configurational change, and new bond breaking or forming through the variation of r_OC_ and r_CN_. As OH^–^ attacks C of a terminal −C≡N (i.e., r_OC_ reduces) of a 4-mer PAN from a close distance (∼3.5 Å) (Figures 2A, 2D), it dissociates from Li^+^ and helps form the first ring by overcoming a barrier (Δ*W*_1_) of ∼9 kcal/mol. This is higher than the barrier for the reverse process (Δ*W*_1_^*R*^∼5 kcal/mol), which is attributed to the gas-phase environment that makes LiOH dissociation, and consequently, the OH^–^ attack on C less probable. However, once the first ring has formed, the barriers for forming the remaining rings (Δ*W*_2_ and Δ*W*_3_) are within 3 kcal/mol, as indicated by the free-energy profiles of r_CN_ (Figure 2B). For the 10-mer PAN, the cyclization barriers for the fifth and ninth units (Δ*W*_5_ and Δ*W*_9_ in Figure 2C) are similarly small (≤3 kcal/mol). Interestingly, regardless of whether it is a 4-mer or a 10-mer chain, the cyclization barrier decreases for each successive unit, suggesting a sequential reduction in the configurational disorder as cyclization advances toward the final unit. Furthermore, the energy barrier for each intermediate cyclization step is lower than that for its reversal, indicating a higher probability of successful sequential cyclization to the final unit once the process begins. An interesting structural change for an intermediate cyclization step (Figures 2E, 2F) as r_CN_ varies is the C–C bond rotation that aligns the −C≡N and −C=N^(−)^ groups on the same side, positioning them close to each other at the transition state and facilitating ring formation. It is also worth noting that the activation energy for purely thermal cyclization is much higher (29–45 kcal/mol)^58^ than that for our room-temperature catalytic cyclization.

**Figure 2:**
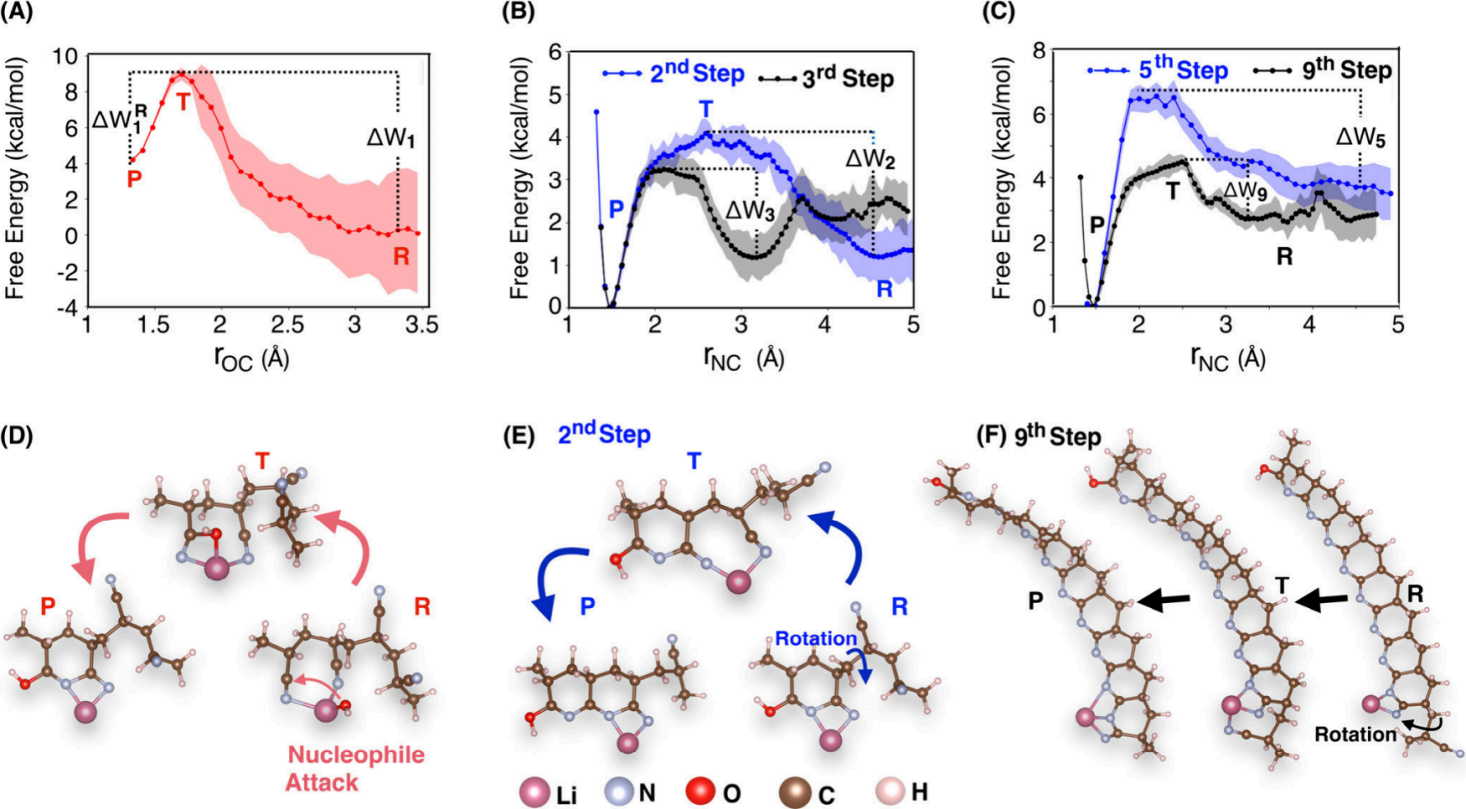
For a 4-mer PAN, the free-energy profiles for the nucleophile (OH^–^) attack (A) and the second and third cyclization steps (B), highlighting the associated barriers, *ΔW*_1_, *ΔW*_2_, and *ΔW*_3_. The same for a 10-mer PAN for the fifth and nineth steps with the barriers, *ΔW*_5_ and *ΔW*_9_ (C). Snapshots illustrating structural changes in going from the reactant (R) to transition (T) to product (P) states of the initial nucleophile attack and second cyclization steps in a 4-mer PAN (D, E) and of the nineth cyclization step of a 10-mer PAN (F).

To quantify the reaction kinetics, particularly to estimate how fast an intermediate cyclization step is, compared to the initialization step, we employ TST within the harmonic approximation

$$k=\left(\frac{k_{B}T}{h}\right)\;\exp\left[-\Delta W/k_{B}T\right]$$

where *k* is the reaction rate for the temperature T and the barrier Δ*W*. *k*_B_ and *h* are respectively the Boltzmann and Planck constants. Given the initial barrier, ∼9 kcal/mol and a maximum barrier, ∼3 kcal/mol for any intermediate step at 300 K, the initialization occurs at a rate of ∼1.7 μs^–1^, while an intermediate step occurs at a rate of 0.0403 ps^–1^ or faster. That is, the latter is 4 orders of magnitude faster. The reader is reminded that sequential cyclization occurs due to the successive attack of the electron localized on N of a −C=N^(−)^ unit onto C of the succeeding −C≡N unit. Therefore, electron transfer through the N atoms during cyclization and the paired transfer of Li^+^ with the electron—*both* should occur on the same fast time scale along an extended polymer configuration.

To provide evidence of electron-coupled Li^+^ transfer during the cyclization process, we perform natural population analysis^59^ on the NNMD snapshots of a 4-mer PAN, representing R, P, and T states. Using a variety of electronic structure methods, we find very similar partial atomic charges across these methods (see SI). Figure 3A shows, from the MP2-level^60^ calculations, how the charge on each N of the first to fourth PAN units changes as the cyclization progresses to the fourth unit. In specific, the negative charge on the fourth N is maintained at ∼−0.5e during the first and second cyclization steps, but it increases to ∼−0.9e after the third cyclization step. In reverse, the ∼−0.9e charge on the second N of the singly cyclized unit reduces to ∼−0.5e after all three units are cyclized. Figure 3B highlights the charges on the N, Li^+^, and nearby C atoms in the product-state snapshots of all three steps. The −0.9e charge confirms the electron localization, i.e., the −C=N^(−)^ group formation, which propagates from one product state to another as the cyclization continues. Interestingly, considering the charges on Li^+^ and its nearest N and C atoms, it appears that a quadrupole-like local configuration, Li^(**+**)^N^(**−**)^C^(**+**)^N^(**−**)^, moves from one cyclized state to another. Going back to Figure 2F, we find that the transition state has a similar configuration, where the dual effects of the nucleophile N^(**−**)^ attacking C^(**+**)^ and the electrophile Li^(**+**)^ attacking N^(**−**)^ are apparent, and are likely what is causing rapid cyclization and charge transfer at any intermediate step.

**Figure 3:**
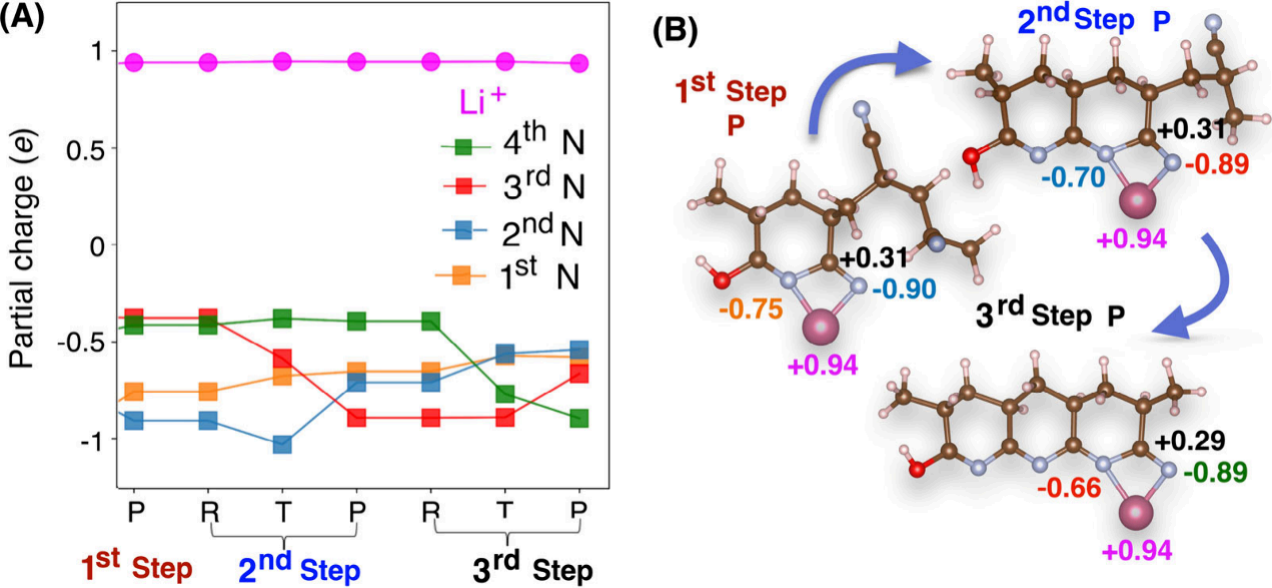
Partial charge of the N atoms in a 4-mer PAN in the R, T, and P states of all three cyclization steps (A). A quadrupole-like configuration is formed in the product state of each step (B).

We recognize that, for any PAN chain-length, a fully extended configuration can make joint nucleophile–electrophile attacks and consequent cyclization most efficient due to minimal configurational barriers. Figures S15–S17 justify this—only a fully extended configuration of a 20-mer PAN, starting with the first-ring cyclized, exhibits nearly complete cyclization and Li^+^ transport to the last unit during a 400 ps-long unbiased NNMD. All other configurations remain stuck in the partially cyclized state. In practice, extended configurations can be accessed by dissolving PAN in polar solvents such as DMF and DMSO, where −C≡N···HC– hydrogen bonds can break rapidly, allowing for efficient reactions (Figure S18). Therefore, we validate our theoretical findings by measuring IR and ^1^H NMR spectra of chain-extended PAN dissolved in a LiOH containing DMSO solution (see SI). Figure 4 summarizes key findings, highlighting effective room-temperature cyclization in the solution state. The increased conjugation is supported by IR analysis (Figure 4A), which shows a progressive decrease in the symmetric −C≡N stretching band (∼2240 cm^–1^) with time, accompanied by the emergence of aromatic (∼1600 cm^–1^) and imine (∼1660 cm^–1^) spectral features. Notably, these positions are slightly blue-shifted in comparison to thermally induced cyclization^61^ which we attribute to the presence of the charged complexes. The DFT-based IR spectra calculations corroborate these findings with the appearance of the −C=N^(−)^ stretching vibration peak at ∼1665 cm^–1^ (Figure S20).

**Figure 4:**
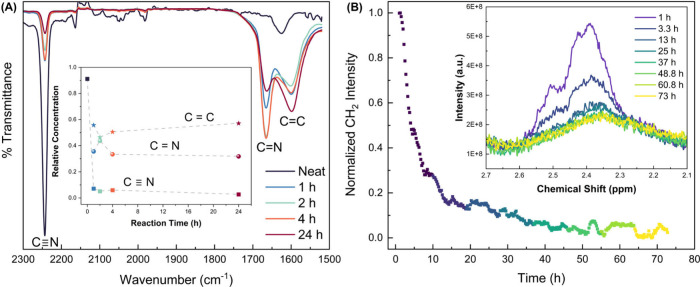
IR spectra of a PAN cyclization products following exposure to LiOH in solution for varying durations and relative fractions of resulting peaks (inset) (A). Decay rate of methylene peak area during in situ ^1^H NMR, indicative of nitrile group consumption. Spectral sampling during the experiment (inset) (B).

To better quantify the kinetics, we formulated a LiOH/PAN mixture in *d*_6_-DMSO and continuously collected ^1^H NMR spectra over the course of several days. The decay of the characteristic methylene groups in the PAN backbone, which are consumed during cyclization and dehydrogenation,^62^ is plotted as a function of time in Figure 4B. A good fit was attained with a biexponential function, suggesting two kinetically distinct phases in the Li-promoted cyclization. This behavior is consistent with ionic initiation, in which rapid cyclization occurs initially in more accessible or prealigned nitrile sequences, followed by a slower phase as reactions propagate into less mobile regions of the polymer where conformational rearrangement and reduced chain mobility limit the rate of further ladder formation.

The computational investigation of cyclization reactions in the bulk PAN, intrachain vs interchain hopping of Li^+^, and ultimately solvent effects is subject to future research. However, the mechanism of electron-coupled Li^+^ ion transport, particularly the propagation of the quadrupole-like local configuration, Li^(**+**)^N^(**−**)^C^(**+**)^N^(**−**)^, along the polymer backbone, should persist in all cases due to the dominant −C=N^(−)^-Li^+^ electrostatics. The current study establishes a deep-learning-enabled computational platform for investigating reactivity and charge transfer in polymers, guiding synthetic chemists in designing polymeric materials for efficient charge conduction in energy storage systems.

## Supplementary Material



## Data Availability

All AIMD data, Gen 7 NNIP model, example lammps script, and PAN chain geometries can be freely accessed at https://github.com/rajnichahal/AIMMS-Research-Group/tree/main/Reactive%20PAN%20NNIP.

## Notes

Comprehensive details of computational methods, experimental procedure, and supporting figures and tables (PDF)

## †

Mechanical and Nuclear Engineering, Tennessee Technological University, Cookeville, Tennessee 38501, USA
