# Supplementary material for: Concerted Electron-Ion Transport by Polyacrylonitrile Elucidated with Reactive Deep Learning Potentials
Source: J Am Chem Soc. 2026 Jun 2;148(23):23457–62. doi: 10.1021/jacs.6c05078 (PMC13281533; doi:10.1021/jacs.6c05078)
Supplement: Supplementary file 1 [file ja6c05078_si_001.pdf]

Supporting Information

Concerted Electron-Ion Transport by Polyacrylonitrile Elucidated with Reactive Deep Learning Potentials

Rajni Chahal-Crockett, Michael D. Toomey, Logan T. Kearney**, Yawei Gao, Joshua T. Damron, Amit K. Naskar, Santanu Roy****

Chemical Sciences Division, Oak Ridge National Laboratory, Oak Ridge, TN-37830, United States

Email: *rchahal@tntech.edu; **kearneylt@ornl.gov; ***roys@ornl.gov

Content: (1) Comprehensive details of Computational Methods (2) Experimental Procedure (3) Supporting Figures (4) Supporting Tables

Abbreviations used in this document: fs = femtosecond; ps = femtosecond; ns = nanosecond; NNIP = Neural Network Interatomic Potential; NNMD = Neural Network Molecular Dynamics; PAN = Polyacrylonitrile; OPLS = Optimized Potentials for Liquid Simulations; DMF = Dimethylformamide; DMSO = Dimethyl Sulfoxide

1. Computational Methods

Here, we describe all steps of the NNIP development, including *ab initio* data generation and the evolution of different generations of NNIP (Gen 0 to Gen 7) based on enhanced sampling and active learning. The results from NNIP-based molecular dynamics, namely, NNMD using the final model (Gen 7), are discussed in the main text. The simulation protocol and the choice of parameters for enhanced sampling molecular dynamics—namely, umbrella sampling and metadynamics—used to compute free energy landscapes are provided. We also discuss the calculations of partial atomic charges using different electronic structure theories to monitor the charge-transfer process during the PAN cyclization steps. Additionally, we detail the all-atom OPLS force field-based MD simulations, which is needed to gain complementary insights into the interaction between PAN and a polar solvent, DMF and how this interaction can potentially affect the PAN cyclization steps.

1.1. Neural Network Interatomic Potential Development

1.1.1. Data generation using Ab-Initio Molecular Dynamics and Umbrella Sampling

We performed the density functional theory (DFT)-based AIMD simulations using the QUICKSTEP module of the CP2K package [1]. We used the double-zeta MOLOPT basis sets (DZVP-MOLOPT) [2] and a 400 Ry cutoff for the plane wave basis, the Goedecker-Teter-Hutter (GTH) pseudo-potential [3], and the Perdew, Becke, and Ernzerhof (PBE) functional [4] to treat the exchange-correlation interactions. Grimme's D3 correction to the dispersion interactions was applied [1], [5]. All AIMD simulations were performed in the canonical ensemble (NVT) using the Nosé-Hoover thermostat [6] with a time constant of 100 fs. In all isolated-chains simulations for 4-mer, 5-mer, 6-mer, and 10-mer PAN chain, the box size of 25 Å, 30 Å, 30 Å, and 50 Å, respectively, were used. However, nonperiodic calculation using the WAVELET Poisson solver for DFT force evaluation were adopted. For bulk partially and fully-cyclized PAN systems (4-mer and 10-mer), the periodic boundary conditions were

implemented, and the system density was varied between 0.124-1.122 g/cc. Using a timestep of 1 fs, the unbiased AIMD simulations were performed for 1.06-5.62 ps for the bulk systems and for 1.47-37 ps for the isolated chain systems. The umbrella sampling simulations in conjunction with AIMD or NNMD to train/retrain NNIP and production run are detailed below. It should be noted that all NNMD runs, whether performed during active learning or production, used an MD integration timestep of 0.1 fs and the Nosé-Hoover thermostat [6] with a time constant of 100 fs to maintain the system temperature at 300 K in the canonical ensemble.

1.1.2. NNIP Training and Evolution the NNIP model

We trained the neural network interatomic potential using the DeePMD-kit (DP-kit) package (version 2.1.1) [26], in which the Deep-Pot-Smooth Edition (DeepPot-SE) was employed to generate a continuously differentiable potential energy surface [27]. The NNIP evolved from Gen 0 to Gen 7, considering both reactive and non-reactive configuration spaces and their necessary enhanced sampling. For generating the configurations along the considered reaction path, namely, reactive configurations, we employed umbrella sampling during AIMD and NNMD simulations by choosing two reaction coordinates, r_{CN} for cyclization variable along the PAN chain and r_{OC} for nucleophile attack, and applying bias potentials with harmonic force-constants of κ_{CN}=100 kcal/mol/Å^2 and κ_{OC}=1000 kcal/mol/Å^2 [7]. Here, r_{OC} is the distance between the hydroxyl (nucleophile) oxygen and the C atom of the first nitrile (−C≡N) group of PAN. r_{CN} is the distance between the N atom of an imino (−C=N$^{(-)}$) group and the C atom of its adjacent −C≡N group. More elaborate details of all training set structures are provided below—specifically, we discuss and illustrate in **Figure S1-S2** how the NNIP evolved from Gen 1 to Gen 7, while our previous work can be consulted for Gen 0 [8]. **Figure S1** points to the inclusion of new structures for training as **Figure S2** depicts the structures stable during each generation. Additionally, we included all the details for training the NNIP models in **Table S1-S8** and their training as well as validation errors in **Table S9**.

Gen. 1. The first generation (**Gen. 1**) of NNIPs was trained on an initial dataset comprising AIMD-equilibrated cyclized structures (r_{CN} =1.5 Å) at 300 K for 4-mer chain with 1 ring (23,650 configurations), 2 rings (26,670 configurations), and all 3 rings cyclized (26643 configurations). In addition, ~8,860 configurations of fully-cyclized 10-mer PAN chains were included in the training as well. To further consider the configurations representing different reaction steps, we initially assumed that the nucleophile OH^- has already attacked the C of the first −C≡N group of the uncyclized 4-mer PAN, creating a −C=N$^{(-)}$ group. Then, we varied the distance between the N atom of this group and the C atom of the next −C≡N group in the range of r_{CN} = 1.3-3.5 Å (0.1 Å interval) to sample a total of ~500 structures for the first cyclization step. Likewise, we collected a set of ~500 structures for the 2nd cyclization step and another set of ~500 structures for the 3rd cyclization step in the 4-mer PAN, where the corresponding r_{CN} was varied between 1.3 Å and 4.8 Å. For sampling specific values of r_{CN}, the umbrella sampling technique was employed with a force constant of 100 kcal/mol. To generate the data set for training, we lift the umbrella bias and run unbiased AIMD for 0.3-1.2 ps using the structures sampled by the umbrella sampling. Although the training errors for Gen. 1 (see **Table S9)** were within the chemical accuracy, only the 4-mer and smaller PAN chains remain stable during the Gen.1-based NNMD simulations (**Figure S2**). For larger PAN chains, unrealistic twists are formed. This instability may be due to insufficient sampling of structures across the reaction paths or due to data imbalance between equilibrated and reactive structures.

Gen. 2. In the 2nd generation, we added additional ~500 configurations, each along the reaction coordinate umbrella-sampled between 1.3-1.4 Å and 1.6-4.8 Å (0.1 Å interval) for 4th and 5th cyclization step for the 5-mer and 6-mer PAN chain, respectively, at 300 K in AIMD simulations. For r_{CN}=1.5 Å, 6,557 4th step on 5-mer and 6,675 5th step on 6-mer chain were sampled at 300 K. Additionally, 11,222 configurations with 1st, 11,717 configurations with 2nd, and 19,515 configurations with 3rd cyclized rings for 4-mer PAN were sampled at 1000 K at the same r_{CN} in AIMD simulations. At same temperature and r_{CN}, 12,610 4th cyclized rings for 5-mer PAN, 3,445 5th cyclized rings for 6-mer PAN, and finally 7,953 fully-cyclized 10-mer PAN chain configurations were sampled to be included in Gen. 2 training. At 1000 K, 1-2 rings opening and reforming were also noticed due to high simulation temperature.

Including this entire dataset in Gen. 2 NNIP stabilized our previously-unstable longer PAN chains (> 4-mer) in NNMD simulations.

Gen. 3. The robustness and reliability of Gen. 2 NNIP was further enhanced by following a standard training-validation-augmentation procedure, where unstable configurations were selected from NNMD simulations followed by DFT calculations for subsequent training. In addition to these, 983 configurations with 1-ring cyclized 4-mer chain and a total of 2162 configurations (523 at r_{CN}=1.5 Å and 1639 at other r_{CN} values) from 2-ring cyclized 4-mer chain and 1376 configurations at r_{CN}=1.5-4.8 Å from 3-rings cyclized 4-mer PAN chains were sampled every 400-1000 fs from 0.53-1.64 ns-long Gen. 2 NNMD trajectories. However, including this dataset in Gen. 3 NNIP training didn't result in any further stability of the NNIP when used for longer, partially uncyclized PAN chains (more H-bonding interactions) in our NNMD simulations.

Gen 4. Further, the Gen. 4 NNIP was trained by adding the DFT data collected from subsampled 4-mer PAN chains by uniformly extracting two sets of ~600 configurations from the stable parts of the 300 ps-long umbrella-sampling NNMD simulations, respectively for the 1st and 2nd cyclization steps. In specific, for the 1st step, a total of ~600 configurations were extracted by biasing r_{CN} at 2.85, 3.35, 5.05 Å, while for 2nd step, another set of ~600 configurations were extracted at r_{CN}= 2.15, 2.95, 3.55, 4.05 Å. All these configurations were labelled in the DFT calculations to be included in Gen. 4 NNIP training. Here, in addition to the previous isolated PAN chain data in Gen. 3, we also added multiple chain pair configurations to enhance the NNIP's stability of longer PAN chains in MD simulations. For this, 8196 configurations of a pair of 4-mer chains and 3266 configurations from triplet of 4-mer chains were included in the training. Furthermore, to improve the NNIP for better accounting for the effect of chain dipole-dipole interactions, we added the entire dataset of Gen. 0 NNIP [8] which comprises 39,156 uncyclized chains and bulk 10-mer and 20-mer chain structures (complete dataset in **Table S1** and in Ref. [8]). The inclusion of these chain pairs, subsampled configurations, and uncyclized PAN dataset drastically improved the stability of the NNIP during simulations of both long PAN chains as well as for NNMD-based umbrella sampling simulations for the free energy surface evaluation of the 1st and 2nd cyclization steps in the 4-mer PAN chain and the 9th cyclization step in the 10-mer chain. However, the Gen. 4 NNIP was still unstable during umbrella-sampling simulations of 10-mer PAN chain during the 1st and intermediate (5th) cyclization steps, where short contacts between atom pairs N-H, N-O, Li-H, etc. lead to breaking of the chain structures. This can potentially be due to still-insufficient representation of configurations, resulting from the dipole-dipole interactions in PAN chains, where large portion of the chain are uncyclized. This can also be due to the lack of distorted bond structures in the Gen. 4 NNIP training.

Gen. 5. In the 5th generation, we further added DFT data from: (i) more subsampled 4-mer chain configurations from the 1st step, namely ~800-860 configurations uniformly sampled from ~1 ns long NNMD simulations at r_{CN}= 2.05, 2.35, 2.75, 3.05, 3.55 Å using umbrella sampling, (ii) 1000-1200 configurations sampled from ~2 ns long NNMD simulations of the 4-mer chain with the 2nd cyclization step at r_{CN}= 2.55, 2.75, 3.25, 4.85, 5.05 Å, (iii) 5618 sheared/distorted triplet with 4-mer PAN chains (1st ring cyclized) where each bond angle is distorted by $\Delta\theta$~0.5 degrees, (iv) ~15,000 equilibrated configurations from AIMD simulations of four Li^+ and four OH^- ions accompanying four 4-mer uncyclized PAN at 800 K, while maintaining periodic boundary conditions (ρ=0.55 g/cc), and (v) bulk periodic structures containing 8 4-mer PAN chains with 1-ring cyclized; namely, 3649 undistorted frames at ρ=0.575 g/cc, 3798 10% expanded (i.e., ρ=0.5207 g/cc), and 5617 5% compressed (i.e., ρ=0.6043 g/cc) configurations. Although this NNIP further stabilized the partially cyclized PAN chains equilibrium configurations for > 500 ps simulations, the long (10-mer) partially cyclized chains were still unstable after ~200-300 ps umbrella-sampling simulations during the 1st and 5th cyclization step.

Gen. 6. To make our NNIP further stable and robust, we develop Gen. 6 NNIP by adding (i) ~150 configurations uniformly sampled from ~1 ns long NNMD simulations at 300 K for the 3rd cyclization step in the 4-mer chain at r_{CN}= 1.85, 3.45, 3.75, 4.65 Å followed by DFT calculations, (ii) 980 configurations each from ~500 ps long NNMD

simulations at 300 K for the 1st cyclization step in the 10-mer chain at r_{CN}= 1.95, 2.35, 3.25, 3.55 Å followed by DFT calculations, (iii) ~37000 total frames collected from running ~500-800 fs long AIMD runs for the 10-mer chain at 500 K. Specifically, independent AIMD runs were carried out by starting with 52 diverse configurations uniformly extracted from ~500 ps long umbrella-sampling NNMD simulations at 300 K while sampling for the 5th cyclization step at r_{CN}= 2.95 Å. Then, we further added (iv) ~8000 frames collected from ~800 fs long AIMD simulations at 300-500 K, which started with 10 diverse configurations uniformly extracted from 10 ps, 380 ps, and 4 ps long NNMD simulations for the 10-mer chain for the 5th cyclization step with r_{CN}=1.85, 3.75, 5.05 Å, respectively. Lastly, we added (v) 1644 periodic bulk structures containing 8 partially-cyclized (1st and 3rd ring) 4-mer PAN chains packed at ρ=0.124 g/cc (1000 K), 1466 configurations packed at ρ=0.9175 g/cc (1000 K), 1061 configurations packed at ρ=1.122 g/cc (1500 K), and 1510 periodic bulk structures containing 8 fully-cyclized 4-mer PAN chains packed at ρ=0.826 g/cc at 1500 K and 1770 periodic structures containing 5 fully-cyclized 10-mer PAN chains packed at ρ=0.512 g/cc at 1500 K. The addition of these structures resulted in a robust NNIP that can allow for sampling across the reaction coordinate for the partially cyclized 10-mer PAN chains, required to the study of the free-energy surfaces of intermediate cyclization steps of partially-cyclized chains.

Gen. 7. At the end, to allow for the nucleophile attack on PAN chain, we used distance between side group C≡N and OH group as reaction coordinate (rc_{OC}) and performed unbiased AIMD simulations (for 2.1-2.3 ps) starting from the structures that were initially optimized at rc_{OC}=1.4-3.5 Å (at 0.1 Å interval) using the Def2-TZVP basis set and PBE D3BJ method [9] in ORCA [10]. From these AIMD simulations, we extracted ~2200 configurations that mostly sampled the reactant (rc_{OC} ~3.5 Å) or product region (rc_{OC} ~1.3 Å) and rarely sampled the transition state region (rc_{OC} ~1.775 Å). Therefore, we decided to add additional configurations by uniformly sampling 2000 configurations using umbrella sampling in Gen. 6-based NNMD simulations (~200 ps long) at rc_{OC}= 1.76, 1.775, 1.78 Å at 300 K. These subsampled structures were further labeled using DFT to compute their corresponding energies and forces for training of Gen. 7 NNIP. **Figure S3-S10** show and compare the energy and forces as predicted by Gen. 1-7 NNIPs in comparison with the DFT predictions for selected validation systems. **Figure S11** shows the comparison of static energy along the reaction path of nucleophile attack on the 4-mer PAN chain. An excellent agreement is observed between Gen. 7 NNIP and ORCA/CP2K calculations. It should be noted that there is a recent surge in foundation models, including MACE models [11], [12] such as MACE-MP-0, MACE-OMAT-0, MACE-MH-0, and MACE-MH-1 that can be used to simulate a variety of materials and molecules. To assess the extent to which different MACE foundation models agree with our findings from our NNIP, in **Figure S11** we also include the static energy along the reaction path of nucleophile attack on the 4-mer PAN chain obtained using the said MACE models. We find that MACE-MH-0 and MACE-MH-1 closely reproduce the energy landscapes from our NNIP, ORCA, and CP2K calculations, whereas the other two foundation models deviate significantly. We attribute the accuracy of MACE-MH-0/1 to the inclusion of a broad dataset involving inorganic crystals, molecules, and surfaces in the training set. However, we anticipate that by fine-tuning MACE-MP-0 and MACE-OMAT-0 with our DFT data, energy landscapes closely resembling those from our NNIP/ORCA/CP2K runs could also be obtained.

In summary, the new configurations and the associated DFT data generation for updating the NNIP from Gen. 1 to Gen. 7 were obtained using the training-validation-augmentation procedure as described above and using the domain-knowledge of this polymer system. The latter guided the selection of sheared configurations and bulk PAN configurations to enhance robustness of the NNIP potential for fully cyclized, partially cyclized, and uncyclized configurations. During all the NNIP training, the datasets were shuffled and split, with 80% and 20% representing the training and validation sets, respectively.

In each training process, the DeepPot-SE model [13], [14] learned a mapping between the local environment of each atom (within 8 Å cut-off) and a per-atom energy, yielding the sum of atomic energies corresponding to the reference DFT energy. Then, the gradients of the NNIP-predicted energies are used to compute

the atomic forces. In training of NNIP, both the reference energies and forces are included to minimize the loss function as training proceeds. The tunable prefactors in the loss function were chosen as 0.002, 1000, 1, 1 for p_e^{start}, p_f^{start}, p_e^{limit}, and p_f^{limit}, respectively. Here, the smooth cutoff and hard cutoff radius of 2 Å and 8 Å is chosen. The embedding network and fitting network sizes are chosen {25,50,100} and {400,400,400}, respectively based on the hyperparameter optimization to yield acceptable final training and validation errors within the precision of DFT energies/forces. **Figure S3-S10** show and compare the parity plots for energies and forces for all the NNIP generations. As such, the low energy and force errors in our Gen 7 NNIP is suggestive of a well-fitted potential energy surface.

In a recent study [15], Kuryla et al. highlighted the importance of quantifying errors in DFT forces arising from the SCF convergence cutoff, as these can have consequential effects on NNIP forces. In all of our AIMD simulations, we chose 1.0E-6 Hartree as the cutoff, higher than default value (1.0E-5) in CP2K and is generally considered a solid, reliable threshold for maintaining energy conservation and accurate forces during AIMD. To ensure that the DFT forces are reasonably accurate, we computed the distribution of per-atom net force along *x*, *y*, and *z* directions, namely, f_x^{net}, f_y^{net}, and f_z^{net} for four different data sets (see below for details). For a closed system (i.e., without an external field), these distributions should peak at zero. Otherwise, the simulated system can unphysically translate/rotate or can even break, which we *do not* observe in our AIMD simulations. Mathematically, we define f_j^{net}, where *j* is *x*, *y*, or *z,* and their norm for a system of *N* atoms as:

$$f_j^{net} = \sum_{i=1}^{N} \frac{1}{N} f_{j,i} \qquad \text{(Eq. S1)}$$

$$|f^{net}| = \sqrt{(f_x^{net})^2 + \left(f_y^{net}\right)^2 + (f_z^{net})^2} \qquad \text{(Eq. S2)}$$

The four different datasets we used are the validation datasets used for parity plots in **Figures S5, S8, S9, and S10**:

Set 1: Bulk 4-mer uncyclized PAN with LiOH at 800 K (**Figure S5**)

Set 2: Bulk 4-mer uncyclized PAN at 1000 K (**Figure S8**)

Set 3: Two 4-mer PAN chains with 1st cyclized step placed at d=1.5 Å (**Figure S9**)

Set 4: OH^- attacking C of CN in 4-mer PAN chain at 300 K, i.e., nucleophile attack with rc = 3.5 Å (**Figure S10**)

In **Figures S22-S25**, we depict the distribution of f_x^{net}, f_y^{net}, and f_z^{net} alongside the distribution of $|f^{net}|$, respectively for **Sets 1-4**. The f_x^{net}, f_y^{net}, and f_z^{net} have nearly symmetric distributions peaking indeed at ~zero and $|f^{net}|$ peaks around 2.5, 1.2, 3.5, and 4.5 meV/Å, respectively for **Sets 1-4.** We note that the component distributions look noisy in **Set 4** due to a significantly smaller number of data points compared to other cases.

1.2 Neural Network Molecular Dynamics
Enhanced Sampling: Umbrella Sampling and Metadynamics

In our umbrella sampling NNMD simulations, a restrained on the r_{CN} (for cyclization variable along the PAN chain) and r_{OC} variable (for nucleophile attack) was implemented by applying a bias potential with a harmonic force-constant of 100 kcal/mol/Å^2. This was achieved by interfacing the PLUMED package [7] with the LAMMPS MD engine. To generate free-energy profiles, along the reaction coordinates (r_{OC} and r_{CN}) at intervals of 0.1 Å, ~0.6-1.1 ns NNMD simulations with an MD timestep of 0.1 fs were performed in the canonical ensemble at 300 K using the final NNIP (Gen. 7). The last 0.5-1.0 ns were used for analysis. Using Grossfield's Weighted Histogram

Analysis Method (WHAM) [16] and a bootstrapping approach, the free-energy profiles were generated along with their errors.

In our metadynamics simulations, a parallel bias well-tempered metadynamics (WT-PBMetaD) was carried out by employing again the PLUMED-LAMMPS MD engine [7]. In performing WT-PBMetaD, one-dimensional Gaussian potentials with widths of $\sigma_{h\text{-bonding}}$ = 0.2 and $\sigma_{dend\text{-to-end}}$ = 0.2 Å were added every 50 steps while keeping the bias factor and the initial Gaussian height of 5 kcal/mol and 3.5 kcal/mol, respectively. Due to nearly twice as efficient NNIP for uncyclized chain as developed in our previous work [8], this NNIP was considered for metadynamics simulations of both 10-mer and 20-mer uncyclized PAN chains. In both simulations, using 10 walkers, different regions in the free-energy landscape during the stretching of uncyclized PAN chain were simultaneously sampled for a total of 18-21 ns using 1 fs time step (i.e., 10 times larger than that used in reactive NNIP-MD simulations). During WT-PBMetaD simulations, two collective variables, h-bonding (CV1) and $d_{end\text{-to-end}}$ (CV2) were chosen. The lower bound of $\sigma_{h\text{-bonding}}$ was chosen to be 0 (no h-bonding) and lower bound of $\sigma_{dend\text{-to-end}}$ was selected to be 0. For $\sigma_{dend\text{-to-end}}$, the upper bound was selected slightly larger than contour length of each PAN chain. In **Figure S14**, we show the free-energy profile of the end-to-end distance for the 10- and 20-mer obtained from these simulations.

1.3 Charge Calculation using Natural Population Analysis

We performed the charge calculation using Natural Population Analysis (NPA) [17] using JANPA package [18], [19]. First, we explored M06 [20](no dispersion), PBE0-D3 [21], PBE0-D3BJ [9], MP2 [22], and SCS-MP2 [23] using Def2-TZVP basis set for evaluating atomic charges for the product state of the 1st cyclization step in the 4-mer chain (reported in **Table S10**). We found only minor changes in charges across these methods. Therefore, we simply chose MP2 for further NPA analyses and evaluated atomic charges for the reactant, transition, and product states of the 2nd and 3rd cyclization steps in the 4-mer chain. The charges for the product states are reported in **Table S11**¸ while charges on selected atoms are shown in the main text. The reactant, TS, and product configurations for each cyclization step were extracted from the NNMD-umbrella sampling simulations performed at their corresponding r_{CN} values.

1.4 Computation of FTIR spectra

Geometry optimizations and subsequent normal mode frequency calculations for the 4-mer PAN in the uncyclized and different cyclized configurations were performed using ORCA [10] at the DFT level using the PBE exchange-correlation functional [4] and D3-BJ dispersion correction [9]. The Def2-TZVP basis set was used [2]. ORCA's inbuilt tool was used to compute broadened FTIR spectra considering an *ad hoc* Gaussian lineshape with a full width at half maximum of 50 cm^{-1}. ChemCraft software [24] was used to visualize vibrational modes and assign the stretching mode frequencies of the –C≡N and –C=N$^{(-)}$ groups.

1.5 OPLS All-Atoms Force Field Simulations

We employed the Large-scale Atomic/Molecular Massively Parallel Simulator (LAMMPS) package [25] to perform MD simulation using the OPLS all-atoms force field [26], [27]. The force field parameters for PAN and DMF are developed using the LigParGen web server [28]. A cutoff distance of 15 Å was used for van der Waals interactions, and 10 Å for Coulomb interactions. Besides, the geometric mixing rules were applied to calculate the van der Waals interactions between different atom types. Long-range Coulomb interactions were computed using the particle-particle particle-mesh (PPPM) algorithm [29] with an accuracy of 10^{-6} kcal/mol/Å.

Table S12 summarizes the composition of the three MD simulation systems. Each system contains folded PAN chain(s) and 7,780 DMF molecules in a cubic simulation box of 100 Å × 100 Å × 100 Å. The PAN chains were initially frozen. Following energy minimization of the DMF solvent, the system was gradually heated from 300 K to 500 K over 1 × 10^5 steps using the NPT ensemble. A Nose-Hoover thermostat and barostat were employed

to control the temperature and pressure, with damping constants set to 100 fs for temperature and 1000 fs for pressure. Subsequently, an NPT simulation at 500 K and zero pressure was performed for 4×10^5 steps with the PAN chains still frozen. This was followed by a temperature ramp down from 500 K to 300 K over another 1×10^5 steps under the same ensemble. To ensure complete equilibration of the DMF solvent, an additional NPT simulation at 300 K was run for 4×10^5 steps. Finally, the PAN chains were unfrozen, and an NVT simulation was conducted at 300 K using the Nose-Hoover thermostat to study the chain unfolding process (**Figure S18**) for 2.5 ns. The MD integration timestep was set to 0.25 fs.

1.6 Umbrella Sampling AIMD Simulations for Initial Free-Energy Surface Evaluation

Prior to developing the NNIP, we intended to achieve a preliminary understanding of the free-energy profiles from AIMD-based umbrella sampling simulations. Considering that the nucleophile attack has already happened, we obtained the free-energy profiles for the r_{CN} coordinate associated with the 1st and 2nd CN units and with the 2nd and 3rd CN units, respectively. Using the same AIMD setting discussed in Section 1.1.1, we further performed AIMD simulations for r_{CN} restrained at 1.2 Å to 3.5 Å at 0.1 Å interval. The harmonic force constant for each simulation was 100 kcal/mol/Å^2. The duration of each simulation for the first step is approximately 40 ps, while the last 30 ps was used for evaluating the free-energy profile by employing the WHAM method [16]. For the second step, the duration of each simulation is approximately 25 ps, while the last 20 ps was used for calculating the free-energy profile. It is worth noting that the purpose of these simulations was compare with and guide the NNMD findings (see **Figure S12**).

We carried out another set of umbrella sampling AIMD simulations to determine a free-energy profile to characterizes Li^+ hopping between the last two units of a fully cyclized PAN 10-mer. We used the same simulation setting as aforementioned. As the reaction coordinate, we chose $\Delta d = d_1 - d_2$, where $d_1$and d_2 are the distances between Li^+ and the carbon atom of the last ($\mathrm{C=N^-}$) and the preceding (C=N) units, respectively. Δd was restrained at -1.15 Å to 1.25 Å at 0.1 Å interval. The duration of each simulation is approximately 20 ps, while the last 10 ps was used for calculating the free-energy profile displayed in **Figure S26**. It appears that the free energy minimum near $\Delta d \approx -0.8$ Å is significantly deeper (approximately threefold) than the minimum near $\Delta d \approx 0.8$ Å from the transition state, indicating that the $\mathrm{C=N^-}$-bound state is thermodynamically more favorable. These depths are the measure of the Li^+ binding free energies at the corresponding binding sites.

We want to point out that the partial charge on the N atom in the neutral C=N unit is similar to that in the nitrile C≡N unit (approximately $-0.5e$; see Figure 3 in the main text). This suggests that Li^+ coordination to neutral C=N and to C≡N should be comparable and, in both cases, substantially weaker than coordination to $\mathrm{C=N^-}$. Consistent with this picture, we emphasize that these site-to-site binding differences and cyclization barriers at intermediate steps are comparable in magnitude and are all much smaller than the barrier (~ 9 kcal/mol) for the cyclization initiation/activation step. Therefore, the initiation step remains the rate-limiting step, and once cyclization is initiated, subsequent reactions and associated transport processes are expected to proceed on faster timescales. However, we acknowledge that bulk and solvent environments can bring in addition effects that will be a subject of future research.

2. Experimental Procedure

LiOH and PAN have poorly matching solubility properties and thus require a specific ratio and balance to achieve a uniform single phase solution condition. A lithium hydroxide–containing mixed solvent was prepared by preparing a 2 wt % lithium hydroxide (LiOH) in water solution. DMSO was gradually added to the aqueous solution to produce a clear, single-phase LiOH/H_2O/DMSO stock solution at a final volume ratio of 3:1 of DMSO to water. Separately, a 1 wt % PAN in DMSO solution was prepared by dissolving a polyacrylonitrile-co-methyl acrylate copolymer with degree of polymerization of ~ 2260 (Sigma Alrich, St. Louis, Missouri). The LiOH containing mixture was then added to the polymer solution to a final molar ratio of 4:1, LiOH to PAN repeat units. The solution was then allowed to react at room temperature for 1, 2, 4 and 24 h prior to precipitation in DI water to stop the

reaction and centrifuged. All samples were dried under vacuum resulting in colored products that were analyzed via ATR-FTIR. The normalized spectra were analyzed within the Origin software environment to extract relative concentrations of characteristic vibrational modes. We present evidence of C–OH formation due to the nucleophilic OH attack in **Figure S27** by highlighting the C–O stretching region, and we demonstrate evidence of sequential cyclization in the main text (**Figure 4**) by highlighting spectral signatures of the imine and nitrile groups.

In-situ nuclear magnetic resonance measurements were constructed in identical concentrations but instead substituting in d6-DMSO for the corresponding mass fraction. ^{1}H NMR experiments were performed on a 400 MHz NEO Bruker spectrometer equipped with a diff50 1H/X BB probe. Due to the complex nature of the sample a lock signal was not possible on the d6-DMSO in the sample requiring manual shimming from the^{1}H FID. ^{1}H data we collected every 15 min using this zg2d pulse sequence. Due to concerns about the drift, the peak areas used for the kinetic analysis were normalized to the residual DMSO peak throughout, though we observed no substantial difference between these values and direct integration.

3. Supporting Figures

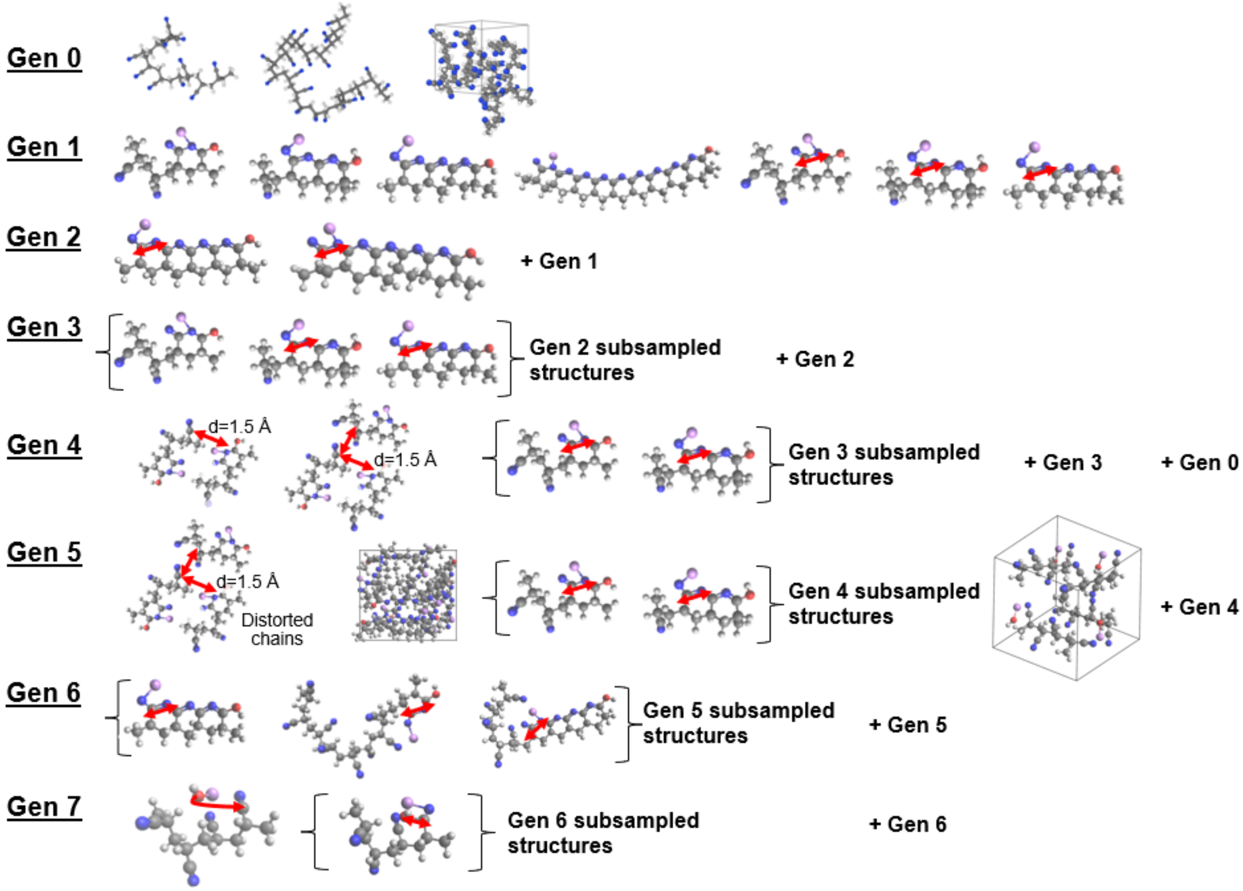

Figure S1. A detailed schematic of training set structures in Gen 0 **[8]** and Gen 1-7 NNIPs developed for PAN polymer. The red double arrow represents sampling across the shown reaction path. More specific details on number of each structure, values of reaction coordinates, and system densities can be found in Table S1-S8.

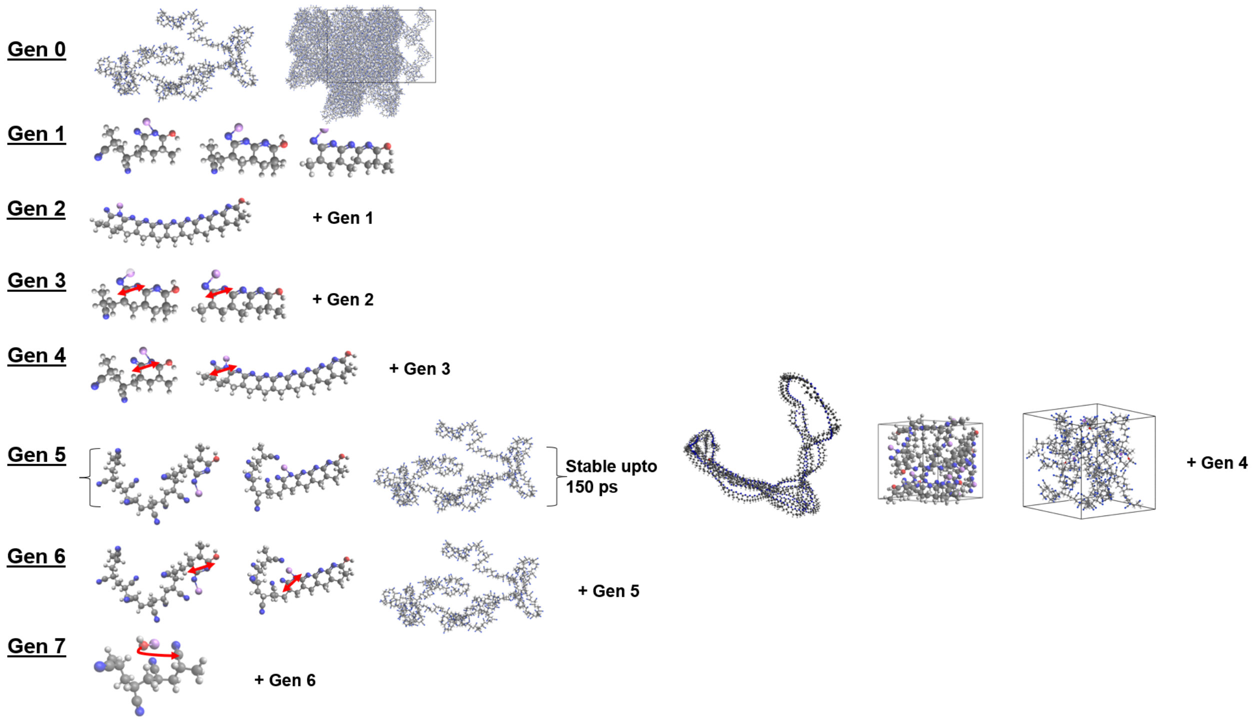

Figure S2. A detailed schematic of stable structures in Gen 0 [8] and Gen 1-7 NNIPs developed for PAN. The red double arrow represents sampling across the shown reaction path.

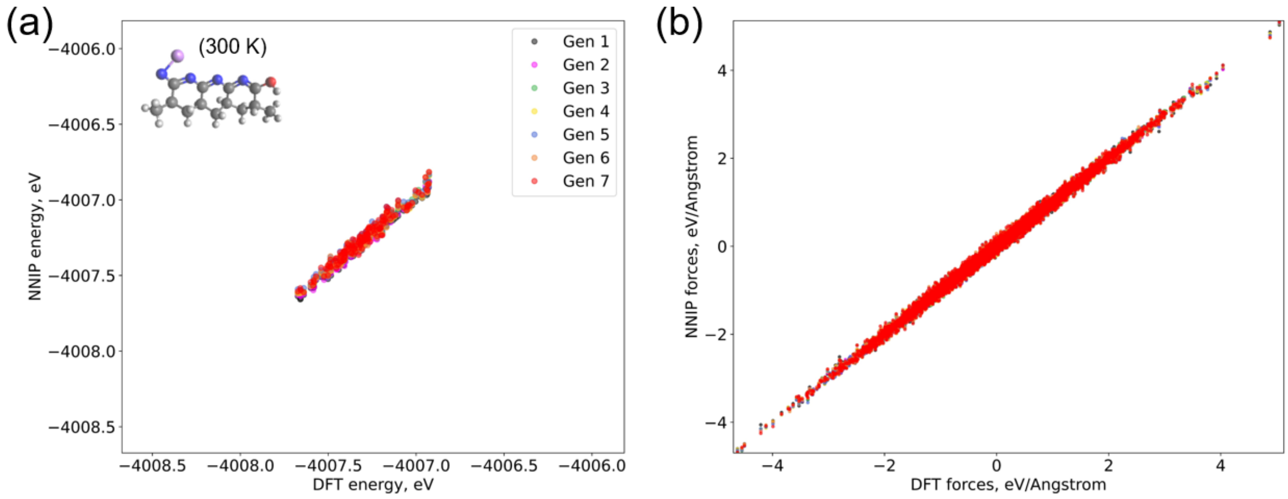

Figure S3. The evolution of the reactive NNIP as reflected in **(a)** energy and **(b)** force parity plots on the validation sets for the system with the fully cyclized 4-mer PAN chain.

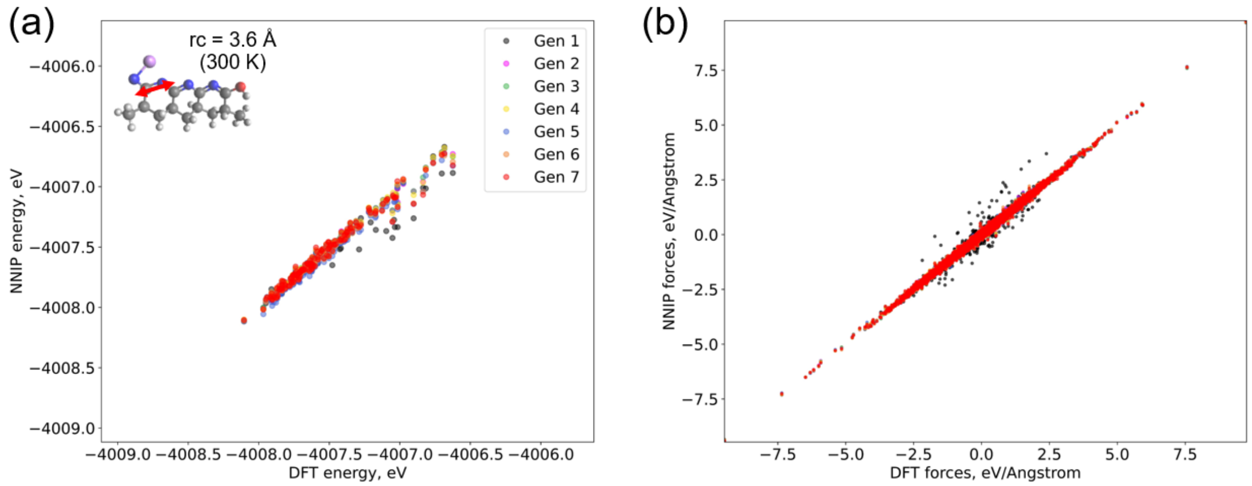

Figure S4. The evolution of the reactive NNIP as reflected in **(a)** energy and **(b)** force parity plots on the validation sets for the 4-mer PAN chain along the reaction coordinate for the 3rd cyclization step.

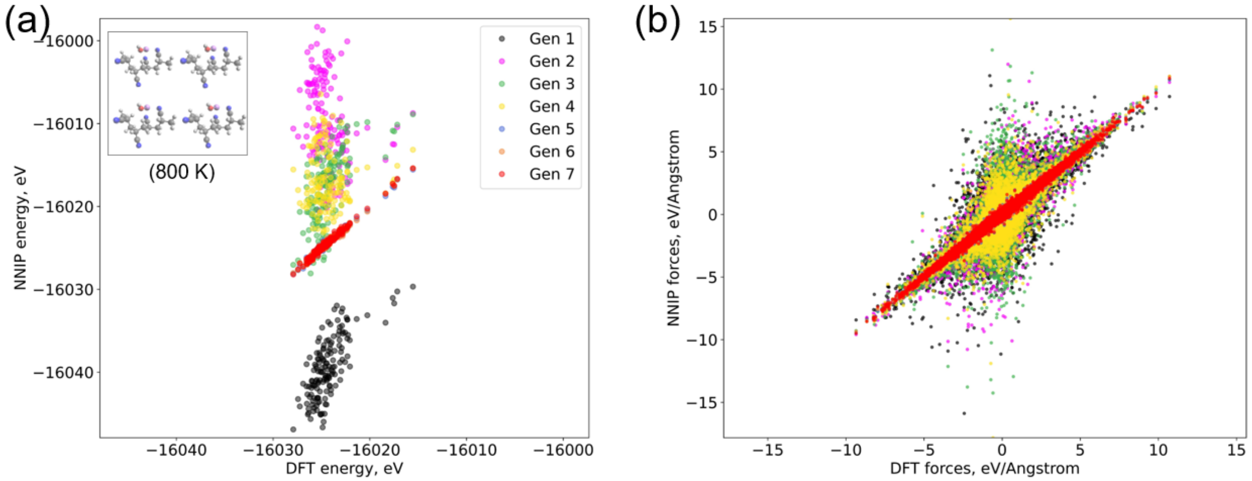

Figure S5. The evolution of reactive NNIP potentials as reflected in **(a)** energy and **(b)** forces parity plots on the validation sets for a system with four units of the 4-mer uncyclized PAN with LiOH. Periodic boundaries were considered in all direction while setting the system density at 0.55 g/cc.

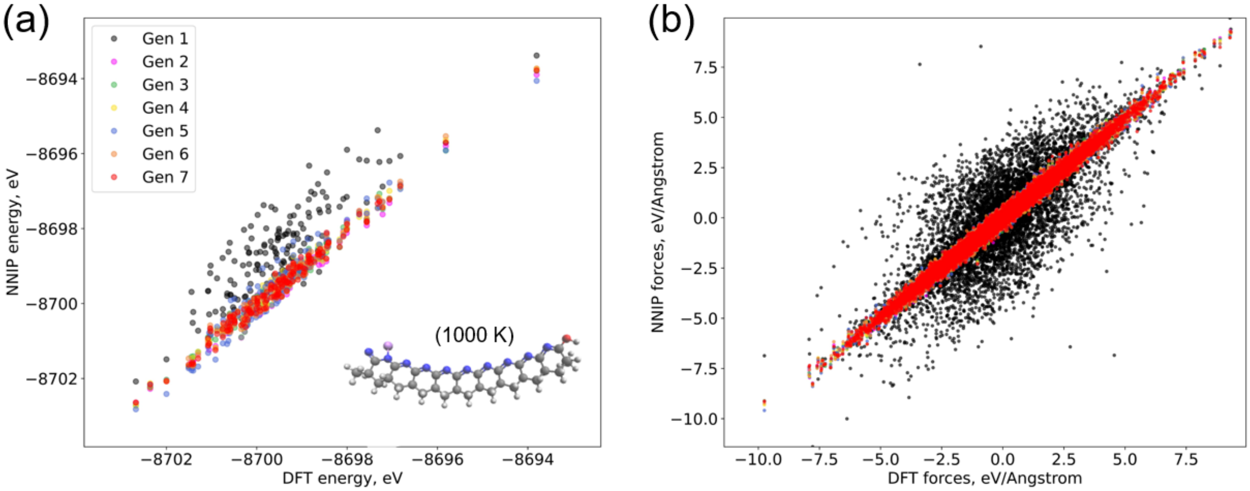

Figure S6. The evolution of the reactive NNIP as reflected in **(a)** energy and **(b)** force parity plots on the validation sets for the system with a fully cyclized 10-mer PAN chain.

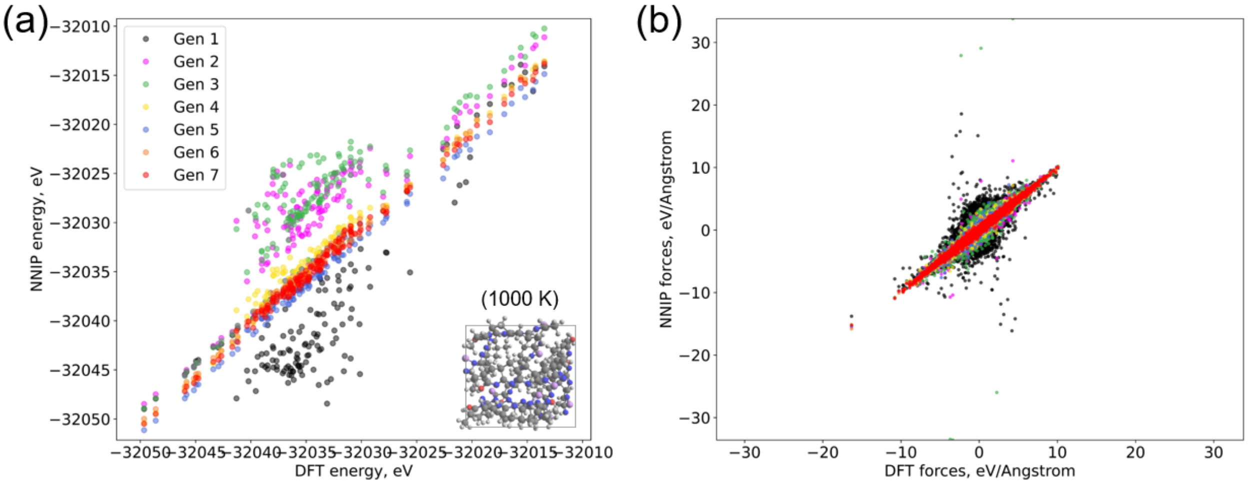

Figure S7. The evolution of the reactive NNIP as reflected in **(a)** energy and **(b)** force parity plots on the validation sets for the system with eight units of 4-mer PAN with the 1st ring cyclized. Periodic boundaries were considered in all directions. The system density was set at 0.1241 g/cc.

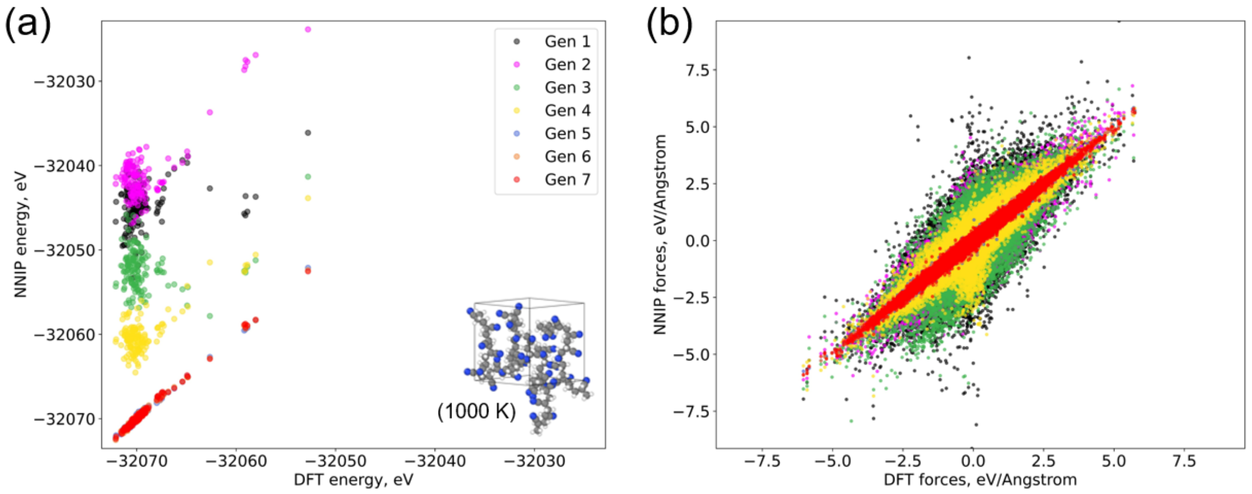

Figure S8. The evolution of the reactive NNIP potential as reflected in **(a)** energy and **(b)** force parity plots on the validation sets for a system with four units of 4-mer uncyclized PAN. Periodic boundaries were considered in all directions. System density was set at average experimental density of 1.195 g/cc **[30]**.

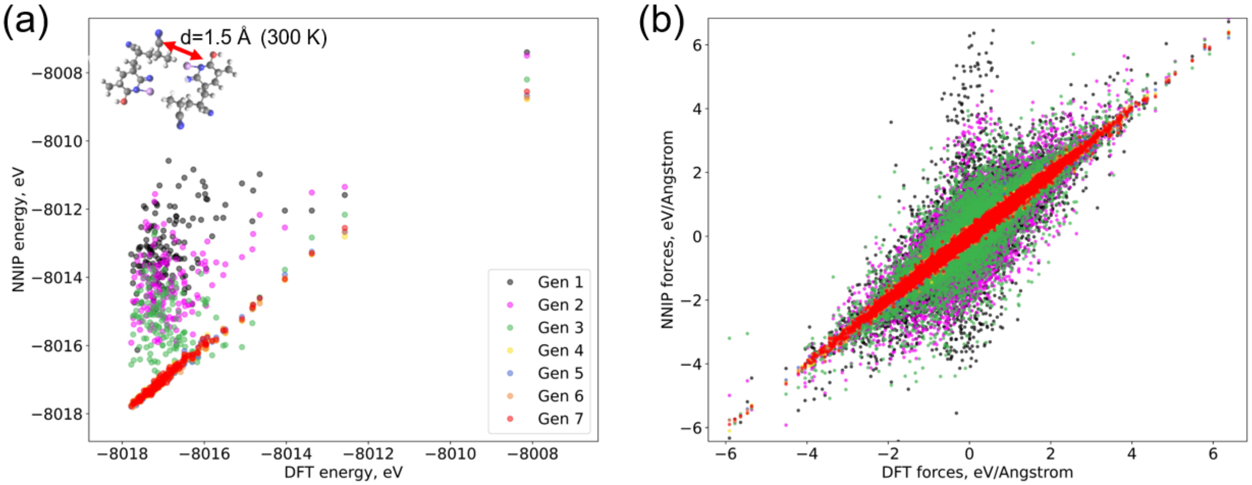

Figure S9. The evolution of the reactive NNIP as reflected in **(a)** energy and **(b)** force parity plots on the validation sets for two 4-mer PAN chain with the 1st cyclized step placed at d = 1.5 Å.

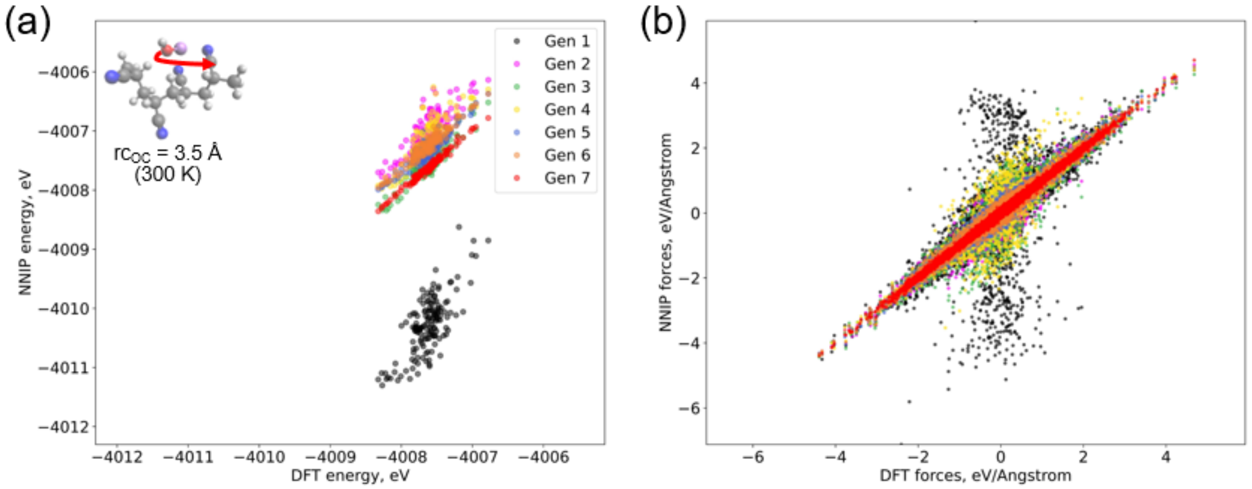

Figure S10. The evolution of the reactive NNIP as reflected in **(a)** energy and **(b)** forces parity plots on validation sets for the system with LiOH (OH^- attacking C of CN) on a fully uncyclized 4-mer PAN chain.

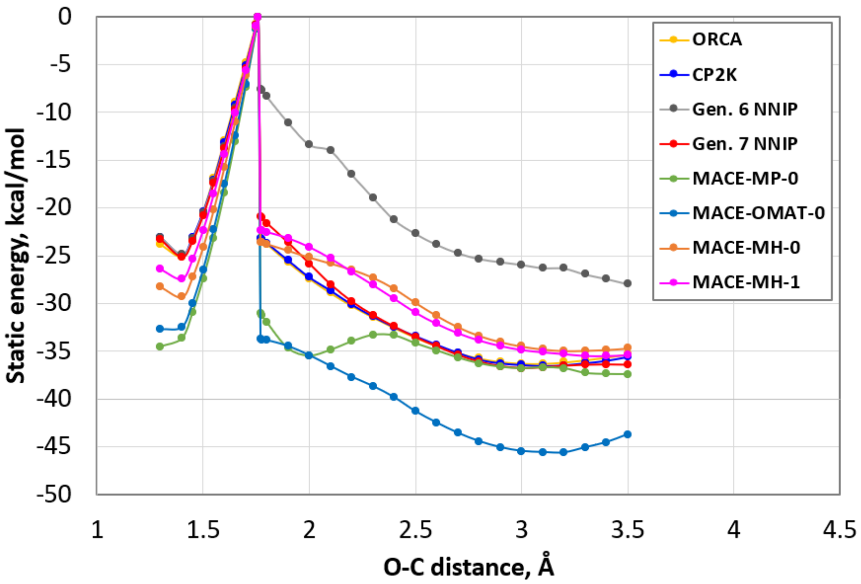

Figure S11. Comparison of the static energies for the DFT-optimized structures during the nucleophile attack on the 4-mer PAN chain: The NNIP-Gen7 energies closely resemble the DFT energies obtained using ORCA (using PBE functional with the D3BJ dispersion correction and the Def2-TZVP basis set) and CP2K (PBE functional with the D3 dispersion correction and the MOLOPT basis set). The comparison with MACE models (MACE-MP-0, MACE-OMAT-0, MACE-MH-0, and MACE-MH-1) is shown as well.

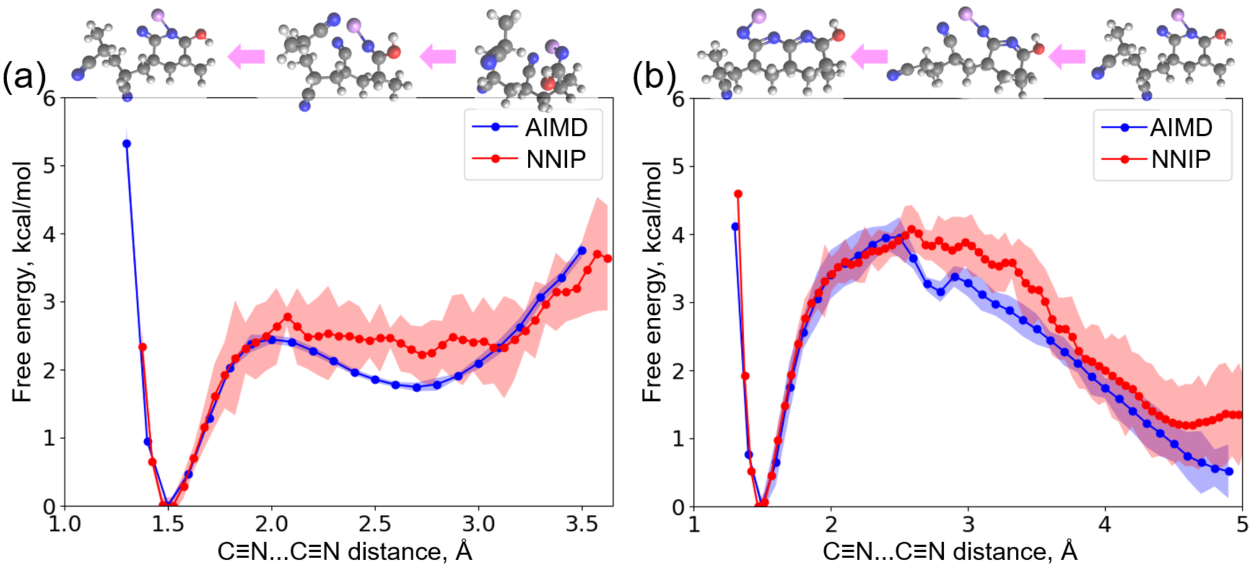

Figure S12. Comparison between the NNIP-predicted and AIMD-predicted free-energy profiles for **(a)** the 1st step considering that the nucleophile attack has already occurred and **(b)** the 2nd cyclization step in the 4-mer PAN chain. Inset depicts the reactant, transition, and product states for both cyclization steps. In the snapshots, N, O, C, H and Li are indicated by blue, red, grey, white, and pink, respectively.

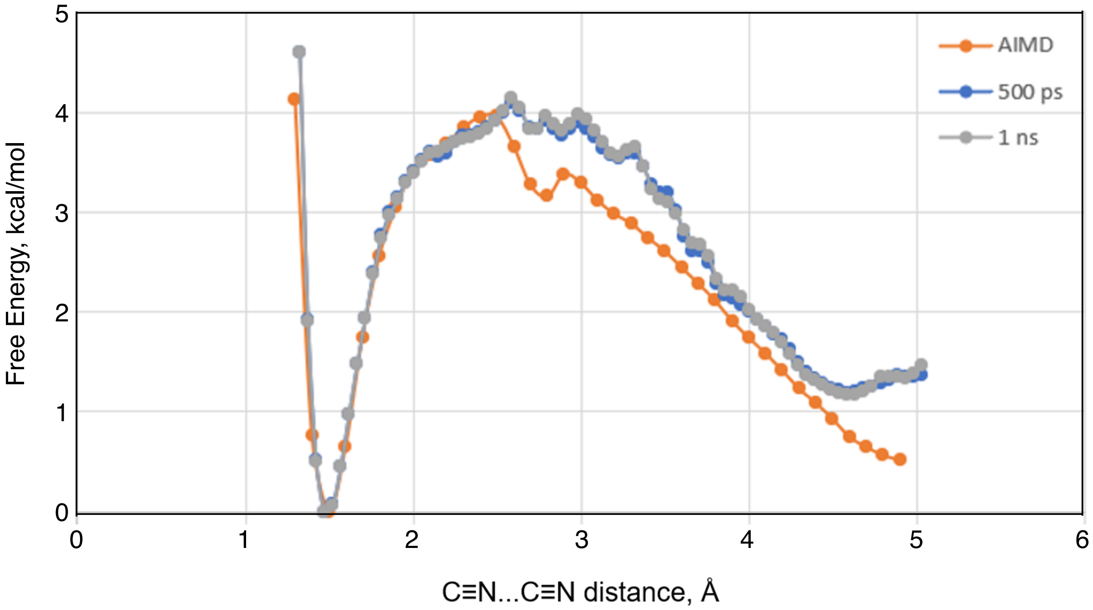

Figure S13. Convergence of the free-energy profiles from the umbrella sampling simulations as shown for the 2nd cyclization step in the 4-mer PAN chain—the 500 ps and the 1 ns NNMD data are identical to each other and are comparable to the AIMD data.

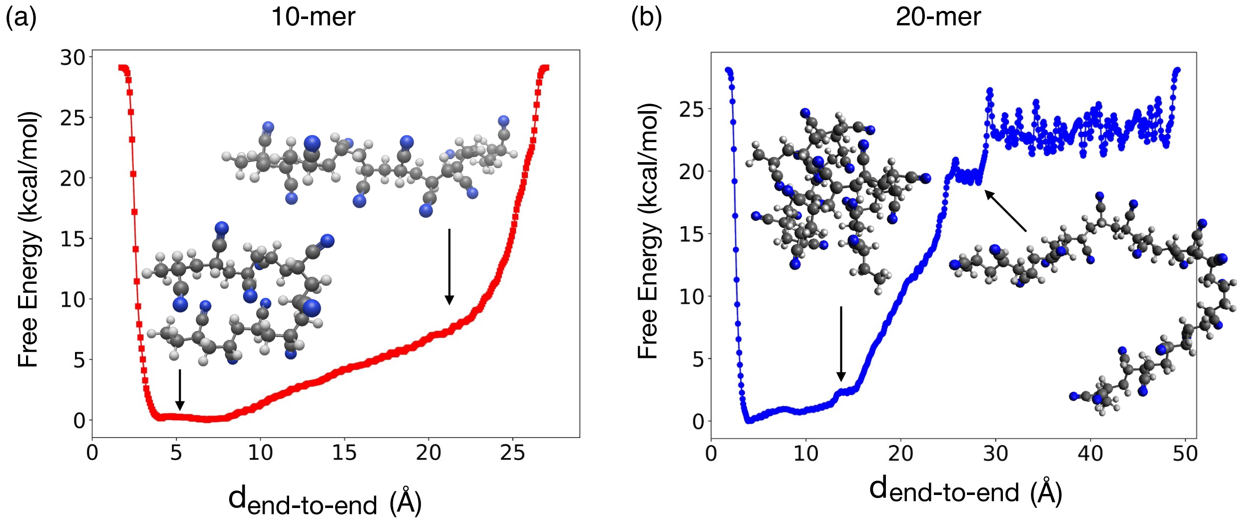

Figure S14. Understanding the free-energy landscape for stretching of (a) 10-mer PAN (b) 20-mer chain as a function of the end-to-end distance obtained using Gen 0 NNIP-based metadynamics simulations. For the 10-mer PAN, the folded and extended configurations can transit without facing a barrier. For the 20-mer PAN, the free-energy landscape is rather rugged, showcasing possible high-energy metastable configurations separated by high barriers. In the snapshots, N, O, C, and H are indicated by blue, red, grey, and white, respectively.

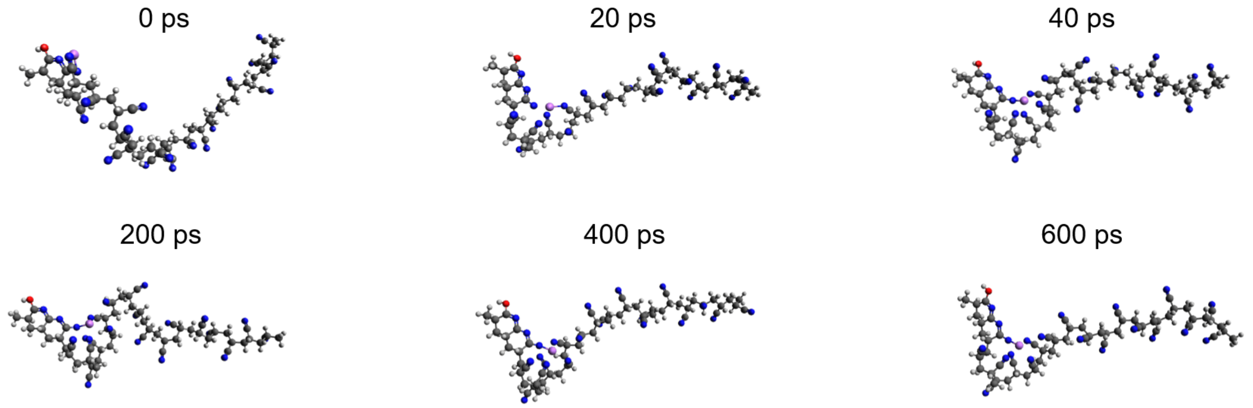

Figure S15. The effect of 73.58% stretching (SR=0.7358) on cyclization of 20-mer PAN chain with 1-ring cyclized at 300 K as predicted by NNIP-based MD simulations. Up to 2 ring cyclization is observed in 1st 20 ps of the simulation, while no further ring cyclization occurs in next 580 ps due to significant h-bonding interactions in remaining uncyclized part of the chain. In the snapshots, N, O, C, H and Li are indicated by blue, red, grey, white, and pink, respectively.

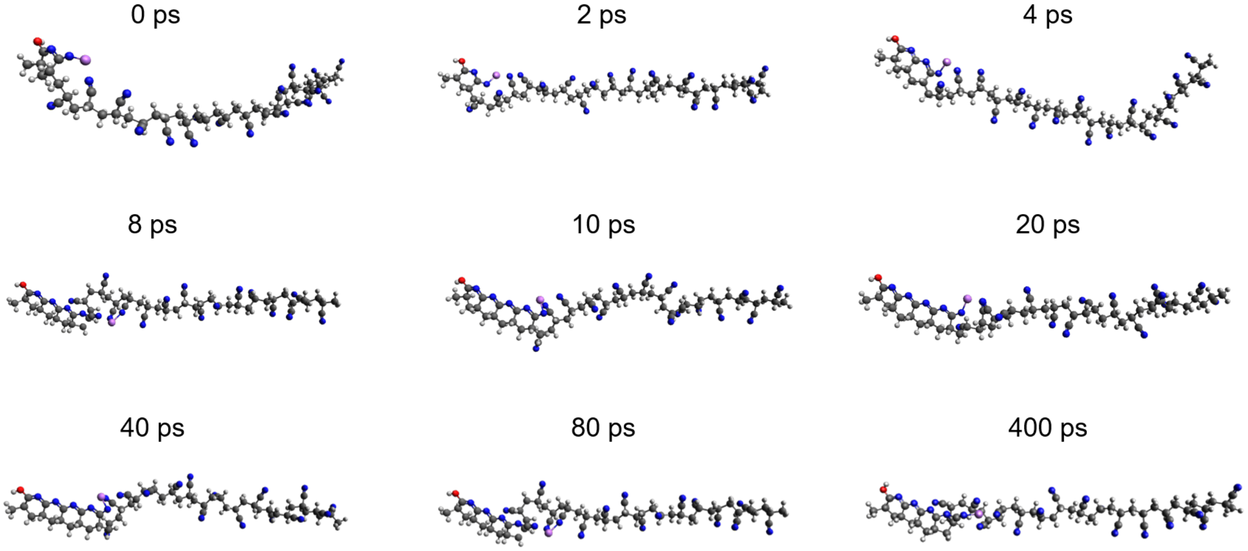

Figure S16. The effect of 95% stretching (SR=0.95) on cyclization of 20-mer PAN chain with 1-ring cyclized at 300 K as predicted by NNIP-based MD simulations. Up to 4 ring cyclization is observed in 1st 10 ps of the simulation, while no further ring cyclization occurs in next 390 ps due to significant h-bonding interactions in remaining uncyclized part of the chain. In the snapshots, N, O, C, H and Li are indicated by blue, red, grey, white, and pink, respectively.

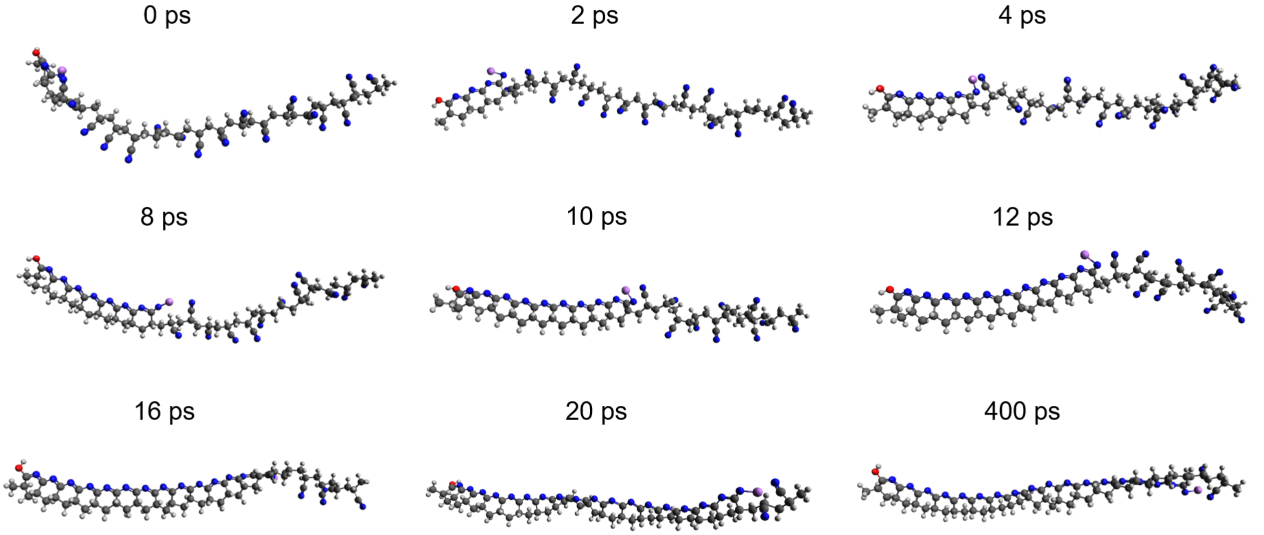

Figure S17. Complete cyclization of the 20-mer PAN chain at 300 K within 20 ps NNMD upon 100% stretching (SR = 1), where the initial configuration has the 1st ring cyclized. Due to high SR value, the h-bonding interactions in uncyclized part of the chain were restricted, which led to significant cyclization in the entire chain within 20 ps. In the snapshots, N, O, C, H and Li are indicated by blue, red, grey, white, and pink, respectively.

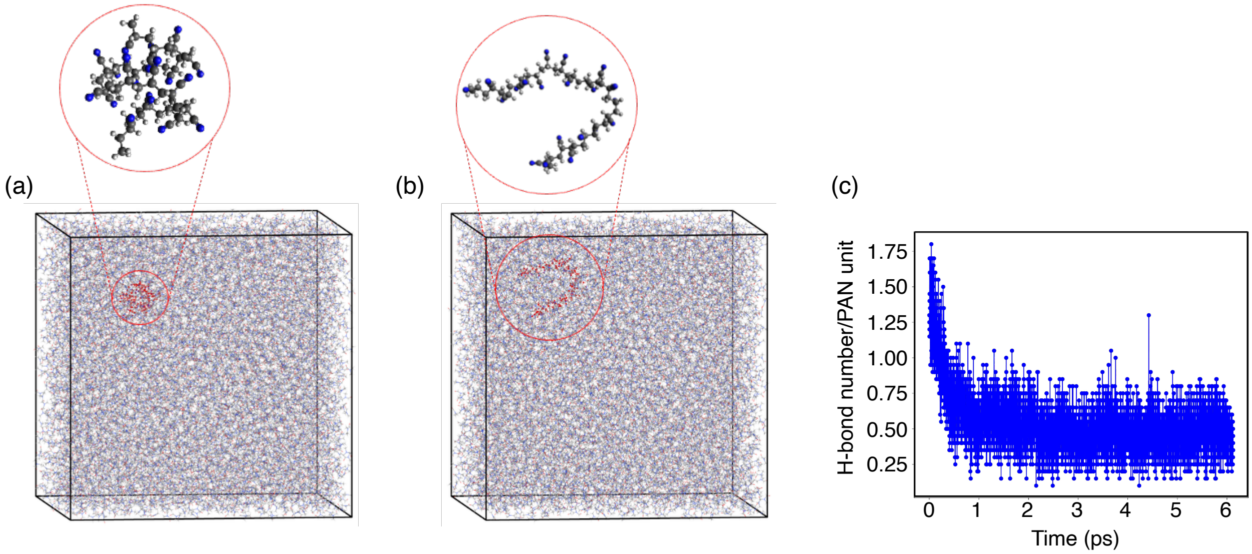

Figure S18. From the OPLS-based MD simulation, the 20-mer PAN chain in the DMF solvent is depicted in the (a) folded and (b) unfolded configurations. It takes about a picosecond for a folded configuration of the 20-mer PAN in DMF to break its intrachain −C≡N·····HC− H-bonds to get to the unfolded or extended configuration. This is gleaned from the time-dependent decay of the H-bond number per PAN nitrile unit of the 20-mer that starts from the folded configuration in DMF (c). In the snapshots, N, O, C, and H are indicated by blue, red, grey, and white, respectively.

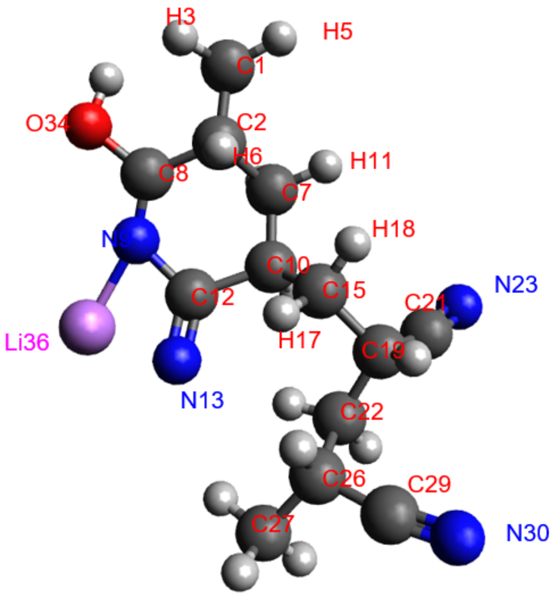

Figure S19. Atom centers marked on a 4-mer PAN chain as reference for the charges reported in **Table S10** and **Table S11.**

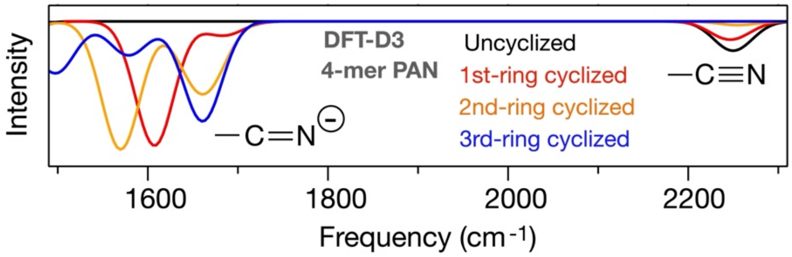

Figure S20. Computed FTIR spectra for uncyclized and different cyclized states of a 4-mer PAN, helping to assign the stretching modes frequencies of the –C≡N and –C=N$^{(-)}$ groups.

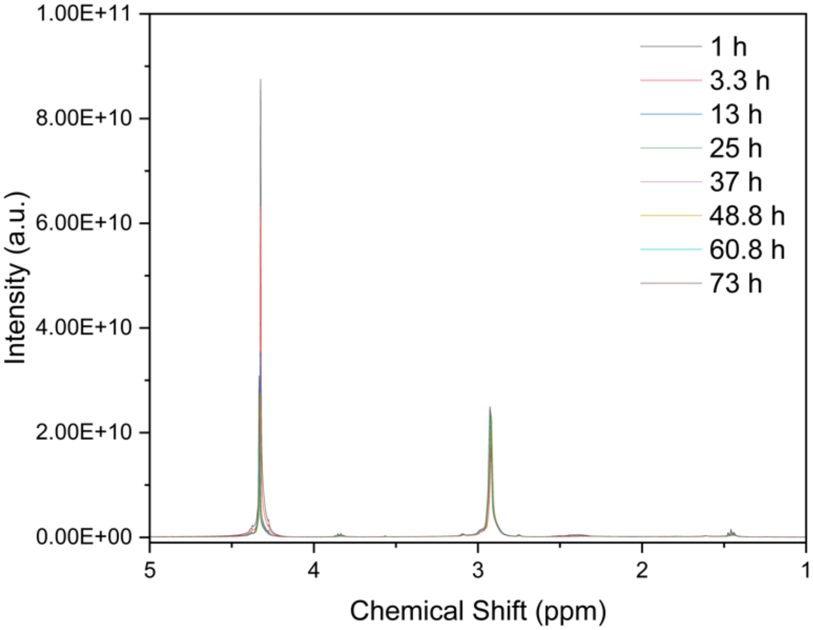

Figure S21. ^{1}H NMR spectra taken at varying times during the in-situ experiment.

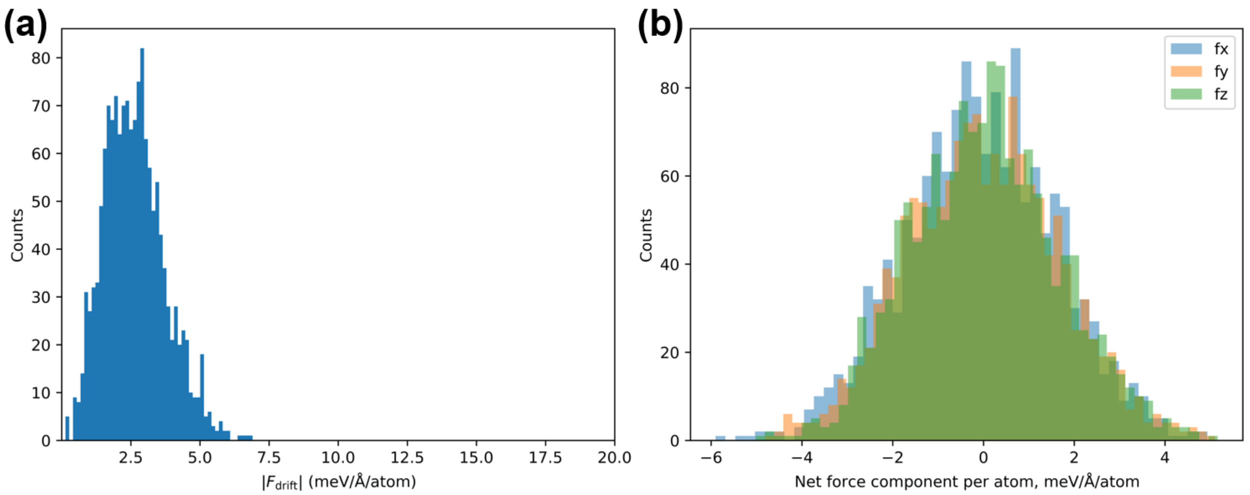

Figure S22. Distribution of per-atom net force: **(a)** $|f^{net}|$ **(b)** f_x^{net}, f_y^{net}, f_z^{net} from the validation dataset used for the parity plot shown in **Figure S5**.

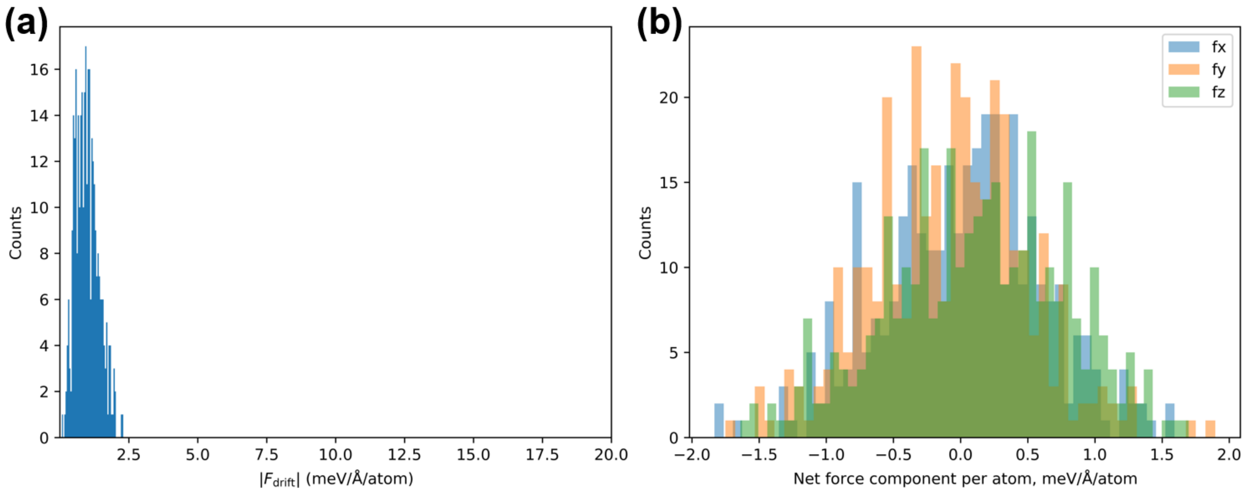

Figure S23. Distribution of per-atom net force: **(a)** $|f^{net}|$ **(b)** f_x^{net}, f_y^{net}, f_z^{net} from the validation dataset used for the parity plot shown in **Figure S8**.

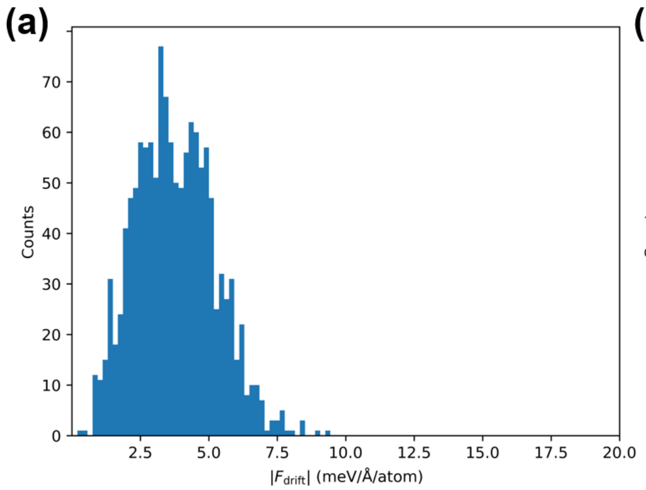

Figure S24. Distribution of per-atom net force: **(a)** $|f^{net}|$ **(b)** f_x^{net}, f_y^{net}, f_z^{net} from the validation dataset used for the parity plot shown in **Figure S9**.

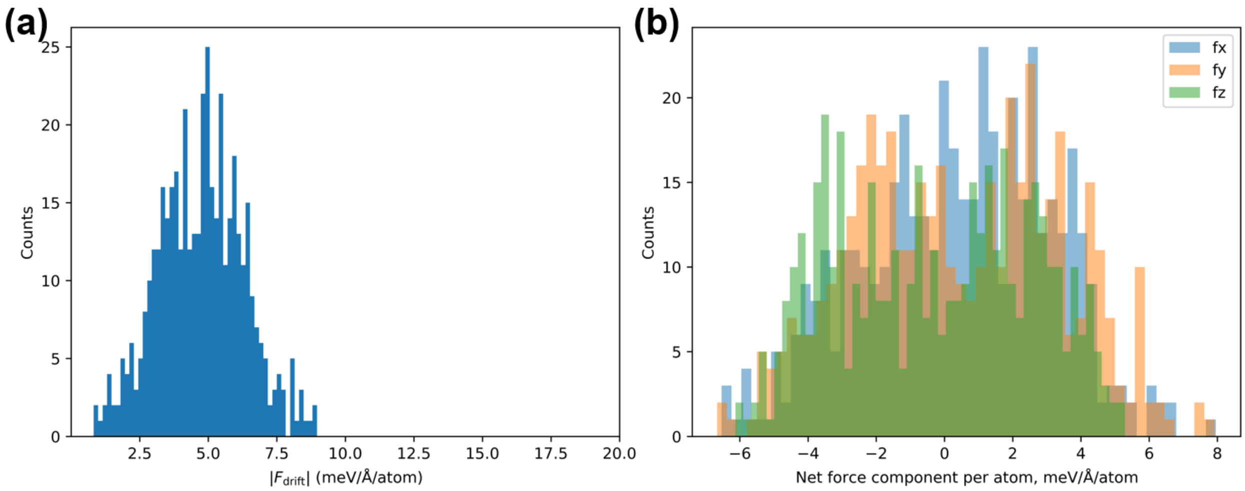

Figure S25. Distribution of per-atom net force: **(a)** $|f^{net}|$ **(b)** f_x^{net}, f_y^{net}, f_z^{net} from the validation dataset used for the parity plot shown in **Figure S10**.

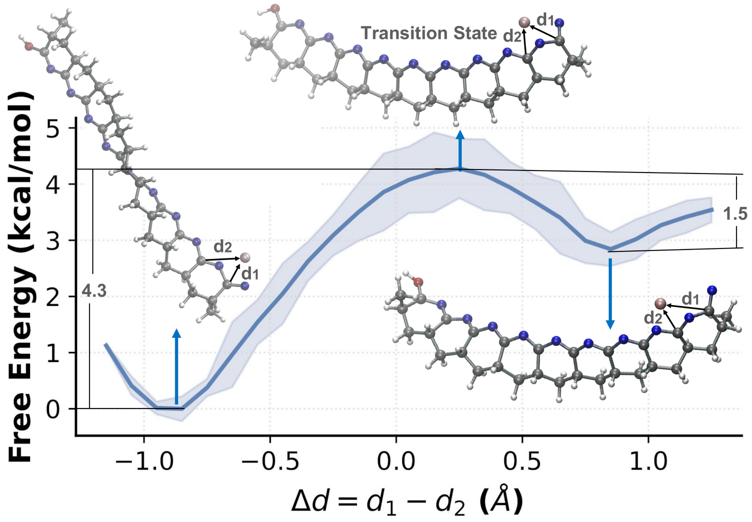

Figure S26. From umbrella sampling AIMD simulations, free energy as a function of Δd, the difference between the distances between Li^+ and C of the last $(C = N^-)$ and its preceding $(C = N)$ units, highlighting a nearly three-times more binding free energy of Li^+ to the last unit than to the preceding unit of a 10-mer PAN chain.

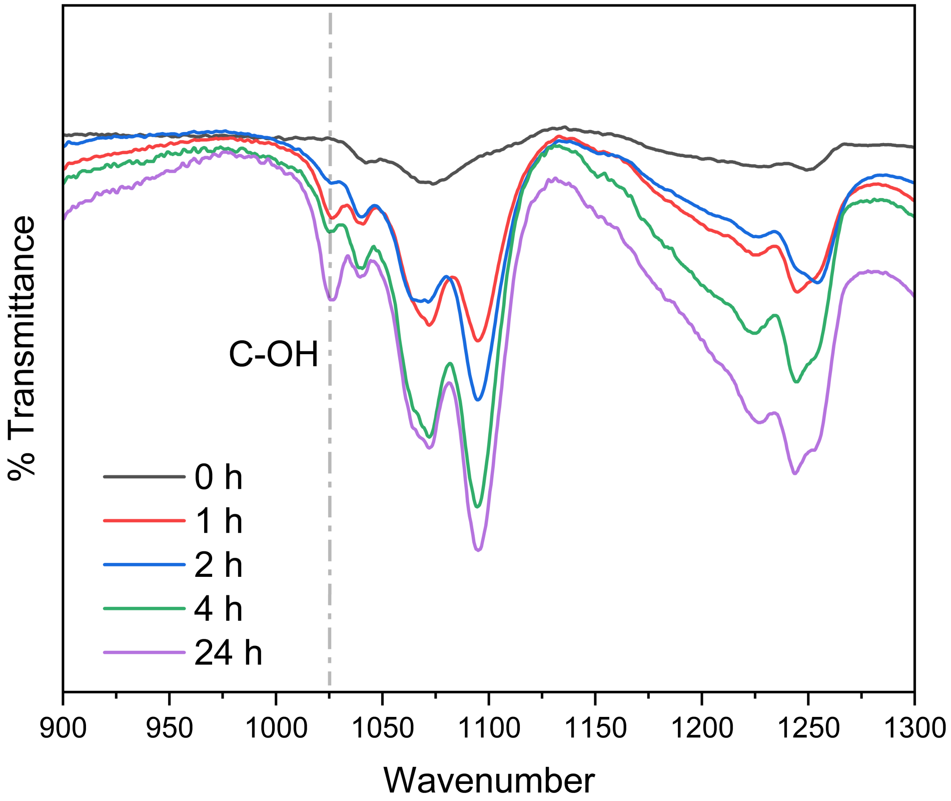

Figure S27. Measured IR spectra of PAN show the emergence of bands around ~1025-1090 cm^{-1}, which can be associated with the C–O stretching region of the C–OH group.

4. Supporting Tables

Table S1. Details of Gen 0 NNIP training.

NNIP	# of relaxed chain configurations		# of bulk configurations				Additional data
	10-mer (T=1000 K)	20-mer (T=1000 K)	10-mer (T=300 K)				
			ρ=1.195 g/cc	-5%	+11%	+34%	N/A
Gen. 0 **[8]**	27324	4368	1722	1580	2013	2149	

Table S2. Details of Gen 1 NNIP training.

NNIP	# of relaxed chain configurations (r_{CN}=1.5 Å)				# configurations along cyclization reaction coordinate (r_{CN})			Additional data
	4-mer (T=300 K)			10-mer (T=300 K)	4-mer (T=300 K)			N/A
	1st	2nd	3rd		1st (r_{CN}=1.3-3.5 Å)	2nd (r_{CN}=1.3,1.4,1.6-4.8 Å)	3rd (r_{CN}=1.3,1.4,1.6-4.8 Å)	
Gen.1	23650	26670	26643	8,860	~500 each	~500 each	~500 each	

Table S3. Details of Gen 2 NNIP training.

NNIP	# of relaxed chain configurations (r_{CN}=1.5 Å)					# configurations along cyclization reaction coordinate (r_{CN})		Additional data
	4-mer (1000 K)	5-mer 4th ring		6-mer 5th ring		5-mer 4th ring (T=300 K) (r_{CN}=1.3,1.4,1.6-4.8 Å)	6-mer 5th ring (T=300 K) (r_{CN}=1.3,1.4,1.6-4.8 Å)	
		T=300 K	1000 K	300 K	1000 K			
Gen.2	11,222 (1st ring), 11,717 (2nd ring), 19,515 (3rd ring)	6557	12610	6675	3445	~500 each	~500 each	NNIP Gen. 1

Table S4. Details of Gen 3 NNIP training. *: Configurations from NNMD subsampling,

NNIP	# of relaxed chain configurations (r_{CN}=1.5 Å)			Additional data
	4-mer (T=300 K)			NNIP Gen. 2
	1st	2nd	3rd	
Gen.3	983*	2162*	1376*	

Table S5. Details of Gen 4 NNIP training. *: Configurations from NNMD subsampling,

NNIP	# of relaxed chain pairs (r_{CN}=1.5 Å)		# configurations along cyclization reaction coordinate (r_{CN})		Additional data
	4-mer (T=300 K)		4-mer 1st ring (T=300 K) (r_{CN}=2.85, 3.35, 5.05 Å)	4-mer 2nd ring (T=300 K) (r_{CN}=2.15, 2.95, 3.55, 4.05 Å)	NNIP Gen.3 + NNIP Gen.0
	2 chains (d=1.5 Å)	3 chains (d=1.5 Å)	~600* each	~600* each	
Gen.4	8196	3266			

Table S6. Details of Gen 5 NNIP training. *: Configurations from NNMD subsampling,

NNIP	# of relaxed chain pairs (r_{CN}=1.5 Å)	# configurations along cyclization reaction coordinate (r_{CN})		Bulk systems with 4 4-mer uncyclized PAN chains with 4 LiOH (800 K)	Bulk systems with 8 4-mer PAN chains with 1 ring cyclized (300 K)	Additional data
	3 distorted chains of 4-mer PAN (T=300 K) (Δθ=0.5 degrees)	4-mer 1st ring (T=300 K) (r_{CN}=2.05, 2.35, 2.75, 3.05, 3.55 Å)	4-mer 2nd ring (T=300 K) (r_{CN}=2.55, 2.75, 3.25, 4.85, 5.05 Å)			NNIP Gen.4
		~800* each	~1000-1200* each	15000 (ρ=0.55 g/cc)	3649 (ρ=0.575 g/cc), 3798 (ρ=0.5207 g/cc), 5617 (ρ=0.6043 g/cc)	
Gen.5	5618					

Table S7. Details of Gen 6 NNIP training. *: Configurations from NNMD subsampling, #: Configurations from subsequent AIMD run.

**NNIP**	**# configurations along cyclization reaction coordinate (r_{CN})**			**Bulk systems**			**Additional data**
	4-mer 3rd ring (T=300 K)	10-mer		**8 4-mer PAN chains with 1 ring cyclized**	**8 fully-cyclized 4-mer chains**	**5 fully-cyclized 10-mer chains**	NNIP Gen.5
		1st ring (r_{CN}=1.95, 2.35, 3.25, 3.55 Å)	5th ring				
Gen.6	~150* each at r_{CN}=1.85, 3.45, 3.75, 4.65 Å	~980* each (300 K)	~8000# each at r_{CN}=1.85, 3.75, 5.05 Å (300 K) ~37000# at r_{CN}=2.95 Å (500 K)	1644 at ρ=0.124 g/cc (1000 K), 1466 at ρ=0.9175 g/cc (1000 K), 1061 at ρ=1.122 g/cc (1500 K)	1510 at ρ=0.826 g/cc (1500 K)	1770 at ρ=0.512 g/cc (1000 K)	

Table S8. Details of Gen 7 NNIP training. *: Configurations from NNMD subsampling,

**NNIP**	**# configurations along nucleophile attack reaction coordinate (rc_{OC})**	**Additional data**
Gen.7	~2200 each at rc_{OC}=1.3-3.5Å for 4-mer chain (300 K)<br>~2000* each at r_{CN}=1.76, 1.775, 1.78 Å for 4-mer chain (300 K)	NNIP Gen.6

Table S9. Average energy and force errors for training (validation) sets as NNIP is updated.

NNIP	Average energy error, eV/atom	Average force error, eV/Å
**Gen. 1**	0.68 (0.67)	45.2 (46.1)
**Gen. 2**	1.07 (1.12)	58.6 (62.3)
**Gen. 3**	1.16 (1.15)	60.2 (62.6)
**Gen. 4**	1.3 (1.34)	64.3 (69.2)
**Gen. 5**	1.65 (1.51)	81.4 (86.9)
**Gen. 6**	1.79 (1.60)	73.2 (79.6)
**Gen. 7**	1.64 (1.52)	76.7 (84.7)

Table S10. NPA charges using various methods on all atoms for the product state of the 1st cyclization step in the 4-mer chain. Atom centers are shown in **Figure S19**.

Center	NPA charge (M06, no dispersion)	NPA charge (PBE0-D3)	NPA charge (PBE0-D3BJ)	NPA charge (MP2)	NPA charge (SCS-MP2)
C1	-0.58887	-0.61487	-0.61488	-0.51794	-0.51207
C2	-0.30153	-0.32217	-0.32217	-0.28669	-0.2689
H3	0.199982	0.208474	0.208477	0.174429	0.168524
H4	0.196683	0.207938	0.207939	0.176414	0.170619
H5	0.232718	0.241776	0.241778	0.213496	0.208517
H6	0.224094	0.231495	0.231497	0.195801	0.193512
C7	-0.38271	-0.39751	-0.39751	-0.32568	-0.32795
C8	0.671008	0.599621	0.599606	0.726833	0.763784
N9	-0.72774	-0.66608	-0.66607	-0.75097	-0.77316
C10	-0.29795	-0.32341	-0.3234	-0.28035	-0.26439
H11	0.208973	0.218143	0.218143	0.185085	0.180349
C12	0.300923	0.265813	0.265827	0.312977	0.34574
N13	-0.85378	-0.83785	-0.83786	-0.90147	-0.92194
H14	0.199105	0.2099	0.209899	0.173669	0.167463
H15	0.228037	0.243278	0.243274	0.206559	0.202887
C16	-0.34962	-0.36749	-0.36749	-0.29764	-0.29061
H17	0.225599	0.235976	0.235975	0.205247	0.198071
H18	0.19399	0.206549	0.206548	0.170717	0.164416
C19	-0.33275	-0.3445	-0.34451	-0.3025	-0.28589
H20	0.246511	0.262783	0.262784	0.229738	0.223435
C21	0.327116	0.287447	0.287442	0.331484	0.329712
C22	-0.38531	-0.39654	-0.39653	-0.33143	-0.32022
N23	-0.34663	-0.32086	-0.32085	-0.37349	-0.37528

H24	0.214104	0.224379	0.224377	0.189327	0.184176
H25	0.246412	0.261069	0.261066	0.234987	0.223391
C26	-0.29666	-0.30609	-0.30608	-0.26612	-0.24397
C27	-0.5768	-0.61221	-0.61221	-0.52017	-0.51033
H28	0.228651	0.244121	0.24412	0.20918	0.203
C29	0.319046	0.277662	0.277668	0.328322	0.329126
N30	-0.37537	-0.34918	-0.34918	-0.40907	-0.41276
H31	0.212426	0.224497	0.224498	0.193318	0.187545
H32	0.19957	0.213734	0.213733	0.182505	0.175313
H33	0.240401	0.250608	0.250605	0.224803	0.218351
O34	-0.71356	-0.66717	-0.66718	-0.72822	-0.75168
H35	0.485128	0.484489	0.484493	0.487249	0.486704
Li36	0.928787	0.926164	0.926165	0.939606	0.93451

Table S11. NPA charges on all atoms for the product states of the 2nd and 3rd cyclization steps in the 4-mer chain using MP2 Def2-TZVP. Atom centers are shown in **Figure S19**.

Center	2nd step	3rd step
C1	-0.514	-0.5646
C2	-0.30506	-0.29306
H3	0.1819	0.20526
H4	0.203	0.19355
H5	0.1822	0.18918
H6	0.2134	0.197
C7	-0.3118	-0.3213
C8	0.7551	0.717
N9	-0.6472	-0.57425
C10	-0.27134	-0.2711
H11	0.186	0.18335
C12	0.539	0.5525
N13	-0.7032	-0.536
H14	0.191	0.1922
H15	0.1831	0.2015
C16	-0.3051	-0.3137
H17	0.17064	0.1766
H18	0.18226	0.1878
C19	-0.27644	-0.2656
H20	0.17133	0.1837
C21	0.31	0.5149
C22	-0.2843	-0.3149
N23	-0.88663	-0.6586
H24	0.20532	0.1637
H25	0.16806	0.167
C26	-0.29253	-0.2377

C27	-0.53664	-0.5043
H28	0.2189	0.1542
C29	0.3356	0.2906
N30	-0.39017	-0.89
H31	0.1809	0.1544
H32	0.184	0.1916
H33	0.2423	0.1964
O34	-0.7156	-0.677
H35	0.4933	0.4744
Li36	0.943	0.9351

Table S12: Compositions of the systems used for OPLS simulations.

	System A	System B	System C
**PAN**	One PAN chain with 10 monomers	Two PAN chains with 10 monomers	One PAN chain with 20 monomers
**DMF**	7780 DMF	7780 DMF	7780 DMF

REFERENCES

[1] T. D. Kühne *et al.*, "CP2K: An electronic structure and molecular dynamics software package - Quickstep: Efficient and accurate electronic structure calculations," *J. Chem. Phys.*, vol. 152, no. 19, p. 194103, May 2020, doi: 10.1063/5.0007045.

[2] F. Weigend and R. Ahlrichs, "Balanced basis sets of split valence, triple zeta valence and quadruple zeta valence quality for H to Rn: Design and assessment of accuracy," *Phys. Chem. Chem. Phys.*, vol. 7, no. 18, pp. 3297–3305, 2005.

[3] C. Hartwigsen, S. Goedecker, and J. Hutter, "Relativistic separable dual-space Gaussian pseudopotentials from H to Rn," *Phys. Rev. B*, vol. 58, no. 7, pp. 3641–3662, Aug. 1998, doi: 10.1103/PhysRevB.58.3641.

[4] J. P. Perdew, K. Burke, and M. Ernzerhof, "Generalized Gradient Approximation Made Simple," *Phys. Rev. Lett.*, vol. 77, no. 18, pp. 3865–3868, Oct. 1996, doi: 10.1103/PhysRevLett.77.3865.

[5] S. Grimme, J. Antony, S. Ehrlich, and H. Krieg, "A consistent and accurate ab initio parametrization of density functional dispersion correction (DFT-D) for the 94 elements H-Pu," *J. Chem. Phys.*, vol. 132, no. 15, 2010, doi: 10.1063/1.3382344.

[6] G. J. Martyna, M. L. Klein, and M. Tuckerman, "Nosé-Hoover chains: The canonical ensemble via continuous dynamics," *J. Chem. Phys.*, vol. 97, no. 4, pp. 2635–2643, 1992, doi: 10.1063/1.463940.

[7] G. A. Tribello, M. Bonomi, D. Branduardi, C. Camilloni, and G. Bussi, "PLUMED 2: New feathers for an old bird," *Comput. Phys. Commun.*, vol. 185, no. 2, pp. 604–613, Feb. 2014, doi: 10.1016/j.cpc.2013.09.018.

[8] R. Chahal *et al.*, "Deep Learning Interatomic Potential Connects Molecular Structural Ordering to Macroscale Properties of Polyacrylonitrile (PAN) Polymer", doi: https://pubs.acs.org/doi/full/10.1021/acsami.4c04491.

[9] S. Grimme, S. Ehrlich, and L. Goerigk, "Effect of the damping function in dispersion corrected density functional theory," *J. Comput. Chem.*, vol. 32, no. 7, pp. 1456–1465, May 2011, doi: 10.1002/jcc.21759.

[10] F. Neese, "The ORCA program system," *WIREs Comput. Mol. Sci.*, vol. 2, no. 1, pp. 73–78, Jan. 2012, doi: 10.1002/wcms.81.

[11] I. Batatia *et al.*, "A foundation model for atomistic materials chemistry," Mar. 01, 2024, *arXiv*: arXiv:2401.00096. Accessed: Apr. 29, 2024. [Online]. Available: http://arxiv.org/abs/2401.00096

[12] I. Batatia *et al.*, "Cross Learning between Electronic Structure Theories for Unifying Molecular, Surface, and Inorganic Crystal Foundation Force Fields," 2025, *arXiv*. doi: 10.48550/ARXIV.2510.25380.

[13] H. Wang, L. Zhang, J. Han, and E. Weinan, "DeePMD-kit : A deep learning package for many-body potential energy representation and molecular dynamics," *Comput. Phys. Commun.*, vol. 228, pp. 178–184, 2018, doi: 10.1016/j.cpc.2018.03.016.

[14] L. Zhang, J. Han, H. Wang, W. A. Saidi, R. Car, and E. Weinan, "End-to-end symmetry preserving inter-atomic potential energy model for finite and extended systems," *arXiv*, no. NeurIPS 2018, pp. 1–11, 2018, doi: https://doi.org/10.48550/arXiv.1805.09003.

[15] D. Kuryla, F. Berger, G. Csányi, and A. Michaelides, "How accurate are DFT forces? Unexpectedly large uncertainties in molecular datasets," *J. Chem. Phys.*, vol. 163, no. 22, p. 224313, Dec. 2025, doi: 10.1063/5.0296997.

[16] A. Grossfield, "WHAM: the weighted histogram analysis method, version 2.0. 9. 2013," *URL Httpmembrane Urmc Rochester Educontentwham Cited On*, p. 35.

[17] A. E. Reed, R. B. Weinstock, and F. Weinhold, "Natural population analysis," *J. Chem. Phys.*, vol. 83, no. 2, pp. 735–746, Jul. 1985, doi: 10.1063/1.449486.

[18] T. Y. Nikolaienko, L. A. Bulavin, and D. M. Hovorun, "JANPA: An open source cross-platform implementation of the Natural Population Analysis on the Java platform," *Comput. Theor. Chem.*, vol. 1050, pp. 15–22, Dec. 2014, doi: 10.1016/j.comptc.2014.10.002.

[19] T. Y. Nikolaienko and L. A. Bulavin, "Localized orbitals for optimal decomposition of molecular properties," *Int. J. Quantum Chem.*, vol. 119, no. 3, Feb. 2019, doi: 10.1002/qua.25798.

[20] Y. Zhao and D. G. Truhlar, "The M06 suite of density functionals for main group thermochemistry, thermochemical kinetics, noncovalent interactions, excited states, and transition elements: two new functionals and systematic testing of four M06-class functionals and 12 other functionals," *Theor. Chem. Acc.*, vol. 120, no. 1–3, pp. 215–241, May 2008, doi: 10.1007/s00214-007-0310-x.

[21] C. Adamo and V. Barone, "Toward reliable density functional methods without adjustable parameters: The PBE0 model," *J. Chem. Phys.*, vol. 110, no. 13, pp. 6158–6170, Apr. 1999, doi: 10.1063/1.478522.

[22] M. Del Ben, J. Hutter, and J. VandeVondele, "Second-Order Møller–Plesset Perturbation Theory in the Condensed Phase: An Efficient and Massively Parallel Gaussian and Plane Waves Approach," *J. Chem. Theory Comput.*, vol. 8, no. 11, pp. 4177–4188, Nov. 2012, doi: 10.1021/ct300531w.

[23] M. Gerenkamp and S. Grimme, "Spin-component scaled second-order Møller–Plesset perturbation theory for the calculation of molecular geometries and harmonic vibrational frequencies," *Chem. Phys. Lett.*, vol. 392, no. 1–3, pp. 229–235, Jul. 2004, doi: 10.1016/j.cplett.2004.05.063.

[24] G. A. Andrienko, "Chemcraft-graphical software for visualization of quantum chemistry computations," *See Httpswww Chemcraftprog Com*, 2010.

[25] A. P. Thompson *et al.*, “LAMMPS - a flexible simulation tool for particle-based materials modeling at the atomic, meso, and continuum scales,” *Comput. Phys. Commun.*, vol. 271, p. 108171, Feb. 2022, doi: 10.1016/j.cpc.2021.108171.
[26] W. L. Jorgensen, D. S. Maxwell, and J. Tirado-Rives, “Development and Testing of the OPLS All-Atom Force Field on Conformational Energetics and Properties of Organic Liquids,” *J. Am. Chem. Soc.*, vol. 118, no. 45, pp. 11225–11236, Nov. 1996, doi: 10.1021/ja9621760.
[27] W. L. Jorgensen and J. Tirado-Rives, “The OPLS [optimized potentials for liquid simulations] potential functions for proteins, energy minimizations for crystals of cyclic peptides and crambin,” *J. Am. Chem. Soc.*, vol. 110, no. 6, pp. 1657–1666, Mar. 1988, doi: 10.1021/ja00214a001.
[28] L. S. Dodda, I. Cabeza de Vaca, J. Tirado-Rives, and W. L. Jorgensen, “LigParGen web server: an automatic OPLS-AA parameter generator for organic ligands,” *Nucleic Acids Res.*, vol. 45, no. W1, pp. W331–W336, Jul. 2017, doi: 10.1093/nar/gkx312.
[29] R. Hockney and J. Eastwood, “Computer simulation using particles taylor & francis,” *Inc USA*, 1988.
[30] R. B. Hurley and L. S. Tzentis, “Density of polyacrylonitrile,” *J. Polym. Sci. [B]*, vol. 1, no. 8, pp. 423–426, Aug. 1963, doi: 10.1002/pol.1963.110010806.
